# Niche shifts and environmental non-equilibrium undermine the usefulness of ecological niche models for invasion risk assessments

**DOI:** 10.1038/s41598-020-64568-2

**Published:** 2020-05-14

**Authors:** Arman N. Pili, Reid Tingley, Emerson Y. Sy, Mae Lowe L. Diesmos, Arvin C. Diesmos

**Affiliations:** 1 The Graduate School, University of Santo Tomas, España, 1015 Manila, The Philippines; 2 HerpWatch Pilipinas, Inc., Tondo, Manila, The Philippines; 3 School of Biological Sciences, Monash University, Clayton, 3800 Victoria, Australia; 4 Philippine Center for Terrestrial and Aquatic Research, Tondo, Manila, The Philippines; 5 Department of Biological Sciences, College of Science, University of Santo Tomas, España, 1015 Manila, The Philippines; 6 Research Center for the Natural and Applied Sciences, University of Santo Tomas, España, 1015 Manila, The Philippines; 7 Philippine National Museum of Natural History, T.F. Valencia Circle, T.M. Kalaw Street, Rizal Park, 1000 Manila, Philippines

**Keywords:** Conservation biology, Ecological modelling, Invasive species, Macroecology, Herpetology

## Abstract

Niche shifts and environmental non-equilibrium in invading alien species undermine niche-based predictions of alien species’ potential distributions and, consequently, their usefulness for invasion risk assessments. Here, we compared the realized climatic niches of four alien amphibian species (*Hylarana erythraea, Rhinella marina, Hoplobatrachus rugulosus*, and *Kaloula pulchra*) in their native and Philippine-invaded ranges to investigate niche changes that have unfolded during their invasion and, with this, assessed the extent of niche conservatism and environmental equilibrium. We investigated how niche changes affected reciprocal transferability of ecological niche models (ENMs) calibrated using data from the species’ native and Philippine-invaded ranges, and both ranges combined. We found varying levels of niche change across the species’ realized climatic niches in the Philippines: climatic niche shift for *H. rugulosus*; niche conservatism for *R. marina* and *K. pulchra*; environmental non-equilibrium in the Philippine-invaded range for all species; and environmental non-equilibrium in the native range or adaptive changes post-introduction for all species except *H. erythraea*. Niche changes undermined the reciprocal transferability of ENMs calibrated using native and Philippine-invaded range data. Our paper highlights the difficulty of predicting potential distributions given niche shifts and environmental non-equilibrium; we suggest calibrating ENMs with data from species’ combined native and invaded ranges, and to regularly reassess niche changes and recalibrate ENMs as species’ invasions progress.

## Introduction

The large-scale redistribution of alien species – i.e., species whose presence in a region is attributed to human activities that enabled them to overcome fundamental biogeographical barriers (*sensu* Richardson *et al*.^1^) – is a defining feature of the Anthropocene^2,3^. Alien species’ invasions can alter the ecology of recipient environments^4,5^ and have socio-economic impacts on recipient jurisdictions^6^. Recognizing these impacts, world nations have committed to develop and implement science-based biosecurity policies and strategies in response to ongoing and future alien species invasions^7,8^, for biodiversity conservation^9^ and sustainability^10^.

Invasion risk assessments assess the ecological and socio-economic impacts of alien species’ invasions, producing the needed information to prioritize alien species and areas for biosecurity intervention^7,11^. Invasion risk assessment activities include predicting the potential distribution (i.e., climatically suitable areas) of invading and/or potentially invasive alien species^11^. These predictions can be made through ecological niche modelling (ENM; aka species distribution modelling)^12–15^ – a correlative statistical tool that quantifies species-environment relationships to define a species’ climatic niche^16–19^. Potential distributions of alien species can then be predicted by projecting ecological niche models across spatio-temporal space, enabling researchers and environmental managers to assess geographical invasion risks and identify areas where an alien species can potentially enter, establish, spread, and cause significant impacts (i.e., susceptible and sensitive sites *sensu* McGeoch *et al*.^20^)^13^. These predictions can, therefore, help in implementing effective biosecurity strategies^15^.

Alien species’ potential distributions are traditionally predicted based on ENMs calibrated using species’ native range data^21^. This approach assumes that species inhabit the entire spatial extent of climatically suitable areas in their native range (i.e., environmental equilibrium)^16,22,23^, and, thus, that native ENMs accurately capture the full extent of a species’ climatic niche^12^. Notably, this approach assumes that species’ climatic niches are conserved across space and time (i.e., niche conservatism *sensu* Wiens and Graham^24^) and, therefore, that species will only occupy areas in recipient regions that are climatically similar to those in their native range^14,21,25^. However, alien species have shown marked climatic niche shifts during invasion (i.e., divergence of climatic niche; *sensu* Broennimann *et al*.^26^), with prevalence varying across taxa (e.g., plants^26,27^, invertebrates^28–30^, and vertebrates^31^ such as amphibians^32,33^, birds^34^, reptiles^32,35^). Climatic niche shifts may be caused by changes in a species’ fundamental niche (i.e., the abiotic limits that define where they can persist; *sensu* Grinnell^36^) due to adaptive changes post-introduction^37^, changes in a species’ realized climatic niche (i.e., the subset of the fundamental niche in which a species can persist subject to dispersal limitations and biotic interactions; *sensu* Hutchinson^38^), or both, due to, for example, enemy release^39^. ENMs calibrated using data from the native range fail to account for climatic niche shifts and, therefore, may underpredict alien species’ potential distributions in invaded regions.

An alternative approach is to calibrate ENMs with data from a species’ invaded range, which, in theory, can capture climatic niche shifts in invading alien species^26,29,40–42^. However, most alien species’ invasions are incomplete (i.e., they have not occupied the full extent of their potential distribution), reflecting environmental non-equilibrium in the invaded range^23,25,41,43^. Moreover, alien species’ invasions in many parts of their invaded ranges are poorly documented or not documented at all^44–46^. To offset limitations of ENMs calibrated using data from native or invaded ranges, past studies have suggested calibrating ENMs using data from the global range (i.e., native and invaded ranges) of an alien species^41,42^, but this approach is also affected by data limitations in the species’ invaded ranges. These limitations of ENMs have raised doubts on their usefulness for invasion risk assessments^23,40–42^.

Quantifying and comparing alien species’ realized climatic niches in their native and invaded ranges provide insight into the extent of niche changes, enabling assessment of niche conservatism and, possibly, environmental equilibrium^26,27,47^. Thus, quantifying realized climatic niche changes can help assess and communicate limitations and uncertainties of ENM predictions, making it a complementary step in alien species’ ENM experiments^23,27,42,47,48^. Realized climatic niches can be quantified and compared in various ways, of which the most widely used methodological frameworks are the unified COUE (i.e., centroid shift, overlap, unfilling, and expansion) framework^47^ and the *n*-dimensional hypervolume framework^49,50^. The COUE framework was purposively developed to assess climatic niche conservatism among phylogenies and species’ populations across space and time^47^. The COUE framework estimates occurrence densities of two entities’ (e.g., species, populations) realized climatic niches in a gridded environmental space to decompose niche changes into three niche metrics: niche stability (i.e., the proportion of the invaded niche overlapping the native niche), niche unfilling (i.e., the proportion of the native niche non-overlapping the invaded niche), and niche expansion (i.e., the proportion of the invaded niche non-overlapping the native niche)^27^. Centroid shift can also be visualized from the topologies of two entities’ occurrence densities in gridded environmental space. Researchers can then compute niche overlap using a similarity index and quantitatively test alternative hypotheses of niche conservatism – niche equivalency tests whether two realized climatic niches in two ranges are equivalent or conserved in the strictest sense, whereas niche similarity tests whether two realized climatic niches are more similar than random niches^48,51^.

An alternative method to quantify realized climatic niches is the *n*-dimensional hypervolume framework^49,50^, which follows Hutchinson’s^38^ description of the fundamental niche. This method first transforms two entities’ climatic or functional (trait-based) niches into hypervolumes within a multidimensional space and, subsequently, compares hypervolumes by calculating indices of hypervolume similarity and metrics of hypervolume distance (centroid and minimum distance) and intersection (volume of intersection, unique fractions). Despite differences in the conceptual theories of the two frameworks, case studies employing both approaches are rare, although one case study of realized climatic niche shifts in an invading alien amphibian species found similar results between the two approaches^33^.

Here we quantified realized climatic niche changes to assess niche conservatism and environmental equilibrium of alien amphibian species in the Philippines and, subsequently, assessed the implications of niche changes for ENM predictions and their usefulness in invasion risk assessments. Six alien amphibian species have been introduced in the Philippines: the green paddy frog (*Hylarana erythraea* [Schlegel, 1837]) in 1880s, the cane toad (*Rhinella marina* [Linnaeus, 1758]) in 1930s, the American bullfrog (*Lithobates catesbeianus* [Shaw, 1802]) in 1960s, the Chinese bullfrog (*Hoplobatrachus rugulosus* [Wiegmann, 1834]) in 1990s, the Asiatic painted toad (*Kaloula pulchra* Gray, 1831) in 2000s, and the greenhouse frog (*Eleutherodactylus planirostris* [Cope, 1862]) in 2010s^52,53^. Pili *et al*.^52^ reviewed their invasion history and updated their current invasion status and distribution in the Philippines. *Rhinella marina* and *L. catesbeianus* were intentionally introduced in the Philippines for biocontrol and food, respectively; meanwhile, *H. erythraea*, *H. rugulosus*, *K. pulchra*, and *E. planirostris* were unintentionally introduced as a contaminant of transported commodities or stowaways on vehicles and cargo^52^. All species except *L. catesbeianus* are now fully-invasive and continue to spread across the country through leading-edge dispersal, as contaminants of transported commodities, and/or as stowaways on vehicles and cargo^52^. The potential ecological and socio-economic changes caused by alien amphibian species invasions^54,55^ emphasize the urgency for researchers and managers to assess the invasion risk of these species, to inform biosecurity^7,11^.

We first quantified and compared the realized climatic niches of *H. erythraea, R. marina, H. rugulosus*, and *K. pulchra* in their native ranges (native niche) and Philippine-invaded ranges (Philippine niche) to investigate climatic niche changes that may have unfolded during their invasion, and to assess niche conservatism and environmental equilibrium. We did not include *L. catesbeianus* and *E. planirostris* because of the limited data on these species in the Philippines. We quantified realized climatic niche changes using both the COUE^47^ and the *n*-dimensional hypervolume frameworks^49,50^. We described species climatic niches and environmental backgrounds using eight environmental variables representing a combination of means, extremes, and seasonality that are known to be ecologically relevant to amphibians and are not highly inter-correlated. We further tested for niche conservatism using niche equivalency and niche similarity tests^48,51^. We then investigated the implications of climatic niche changes for niche-based predictions of species’ potential distributions by calibrating ENMs using data from the Philippine-invaded (Philippine ENMs) and native ranges (native ENMs), and both ranges combined (combined range ENMs). Recognizing that different ENM approaches can yield varying predictions of potential distributions despite being calibrated with the same data (e.g., presence, absence/pseudo-absence, and environmental variables), we quantified ENMs of the four alien species using eight statistical approaches. We then created ensemble models (EMs) for each set of ENMs and used these to predict the species’ potential distributions in the Philippines and in their respective native ranges. Ensemble models identify the signals (i.e., high agreement among models) and filter the noises (i.e., high disagreement) from different ENMs, yielding a lower mean error than any of the constituent ENMs and their resulting predictions^56^. We related observed niche changes to aspects of the species’ invasion history in the Philippines^52^ and to predictions of potential distributions. We conclude with a discussion on the implications of our findings for invasion risk assessments.

We focused our niche shift analyses on the Philippines because we have a relatively robust species’ occurrence dataset on the distribution of the alien amphibians in the Philippines^57^. Data in other invaded ranges of these species are limited, especially for *H. erythraea*, *H. rugulosus*, and *K. pulchra* (data on the invaded range of *R. marina* is similarly poor, with the exception of Australia^33^). Including other invaded ranges in the analysis would therefore poorly represent the species’ global invaded niches. Importantly, including data from additional jurisdictions in which these species are alien would unlikely change our conclusions, because these additional data would be swamped by the higher number of Philippine records^57^.

## Results

### Varying levels of realized climatic niche change

**Figure 1:**
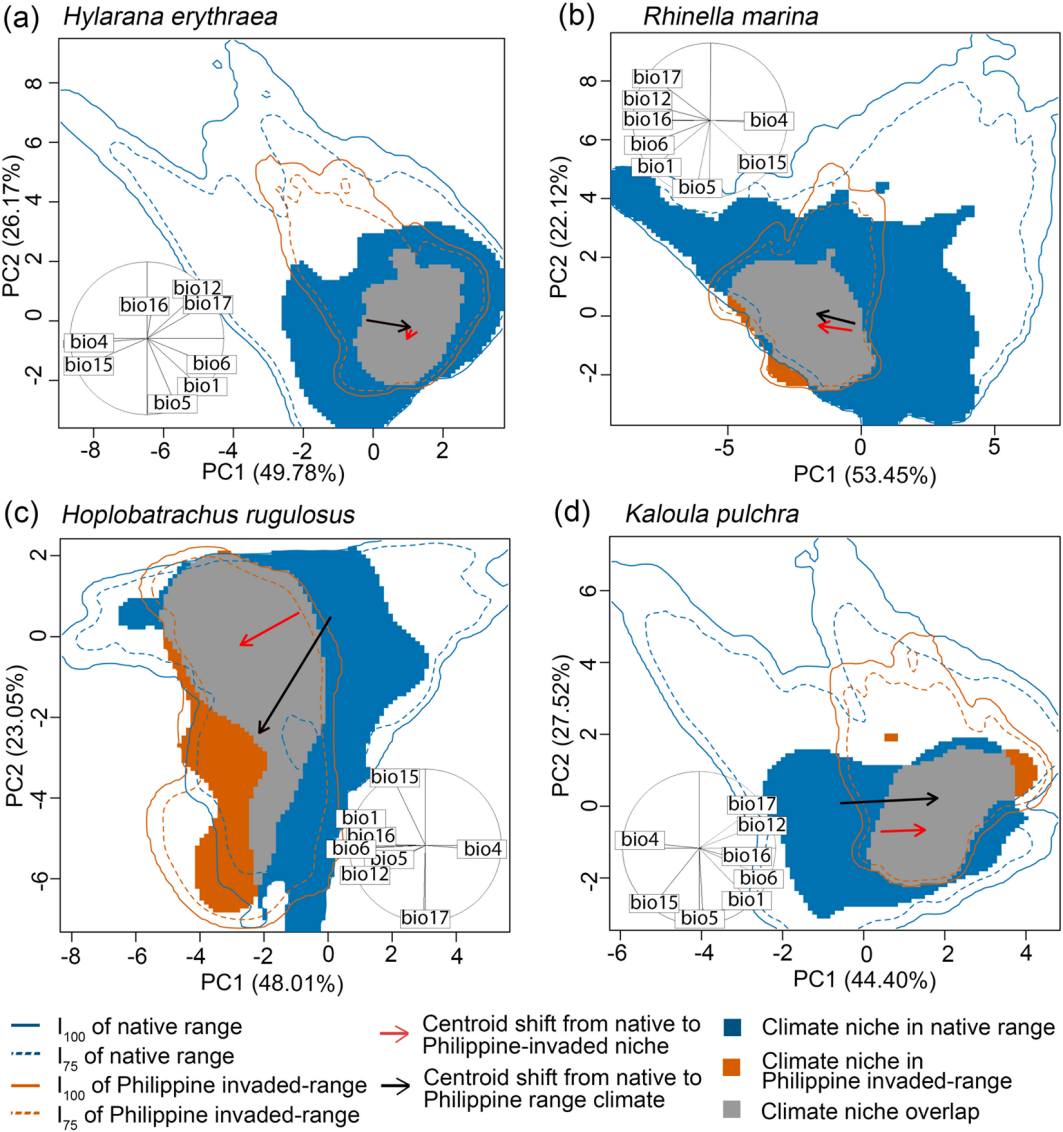
The native (blue) and Philippine (red) niches of *Hylarana erythraea* (**a**), *Rhinella marina* (**b**), *Hoplobatrachus rugulosus* (**c**), and *Kaloula pulchra* (**d**) as depicted by the biplot of the first two PCs. Grey areas represent overlap of the species’ native and Philippine niches. The solid and dashed contour lines respectively represent the intersection of 75% (I_75_) and 100% (I_100_) of available environments in the native range (blue) and in the Philippine-invaded range (red). Solid arrows point to the direction of centroid shift from the species’ native niches’ occurrence density centroid to the that of Philippine niche. Dashed arrows represent the distance and direction between the positions of the centroids of the occurrence density of the environmental backgrounds in the native range and the Philippines along the PC biplots. The correlation circle shows the distribution of eight environmental variables along with the PC biplot: bio 1 = annual mean temperature; bio 4 = temperature seasonality; bio 5 = maximum temperature of warmest month; bio 6 = minimum temperature of coldest month; bio 12 = annual precipitation; bio 15 = precipitation seasonality; bio 16 = precipitation of wettest quarter; bio 17 = precipitation of driest quarter.

Following the COUE framework^47^, we first quantified the occurrence densities of the native and Philippine niches in a weighted biplot of the first two principal components (PCs) of a Principal Components Analysis (PCA) calibrated using pooled environmental backgrounds in the species’ native and Philippine-invaded ranges. The first two PCs captured ~71–76% of the variation in the environmental data. Supplementary Table S1 (online) shows the correlations between environmental variables and PCs. The topology of occurrence densities across the species’ native and Philippine niches in PC biplots revealed: that the Philippine niche of *H. erythraea* is a subset of its native niche, whereas the other species showed partial overlap between native and Philippine niches^58^; high to complete niche stability for all species at the intersection of the 75^th^ percentile of environmental backgrounds (I_75_; 93–100%) and at the intersection of the complete environmental background (I_100_; 91–100%); low to moderate niche unfilling for all species at I_75_ (6–24%) and at I_100_ (13–30%); and zero to low niche expansion for all species at I_75_ (0–7%) and I_100_ (0–9%) (Fig. 1; Table 1). *Rhinella marina* and *H. rugulosus* showed niche expansion into non-analogous environmental space (i.e., climates found in the Philippine environmental background but not in the species’ native range environmental background). *Rhinella marina, H. rugulosus*, and *K. pulchra* shifted their niche centroids to warmer and wetter climates in the Philippines (negligible centroid shift in *H. erythraea*; Fig. 1; see also Supplementary Figs. S1–S4).

**Table 1:** Summary results of three niche change metrics (stability, unfilling, and expansion), overlap index (Schoener’s *D*), and niche equivalency and similarity tests (*P* values). I_75_ and I_100_ represent respectively the intersection of 75% and 100% of available environments in the native and Philippine-invaded range.

	*Hylarana erythraea*		*Rhinella marina*		*Hoplobatrachus rugulosus*		*Kaloula pulchra*	
**Niche change metrics**	*I*_*75*_	*I*_*100*_	*I*_*75*_	*I*_*100*_	*I*_*75*_	*I*_*100*_	*I*_*75*_	*I*_*100*_
*Stability* (%)	100	100	100	100	93	91	98	98
*Unfilling* (%)	24	30	7	13	6	16	10	18
*Expansion* (%)	0	0	0	<1	7	9	2	2
**Overlap** (Schoener’s *D*)	0.13		0.10		0.10		0.26	
**Equivalency test** (*P value*)	1.00		0.001		1.00		0.01	
**Similarity test** (*P value*)	*N* ↔ *P*	*N* → *P*	*N* ↔ *P*	*N* → *P*	*N* ↔ *P*	*N* → *P*	*N* ↔ *P*	*N* → *P*
	0.06	0.14	0.06	0.16	0.42	0.76	0.01	0.01

N ↔ P and N → P represent the randomization methods employed in the niche similarity test.

### Niche equivalency and similarity tests

Species’ native and Philippine niches showed low niche overlap (Schoener’s index of niche overlap [*D*]) (Table 1), with the lowest overlap in *H. rugulosus (D* = 0.10) and the highest in *K. pulchra* (*D* = 0.26). Comparing observed niche overlap values to null distributions (i.e., overlap values estimated from niches constructed by randomly re-allocating pooled occurrences to both niches) revealed that native and Philippine niches were more similar than expected by chance for *R. marina* and *K. pulchra* (*P* ≤ 0.01; Table 1; see Supplementary Fig. S5). Meanwhile, niches were more similar than randomly generated niches only for *K. pulchra* (*P* = 0.01 for both randomization methods). There was also a trend toward the niches of *H. erythraea* and *R. marina* being more similar than randomly generated niches for one of the two types of niche similarity tests (*P* = 0.06 for N ↔ P randomization test). For the case of *H. rugulosus*, niche equivalency was rejected, whereas the results of the two niche similarity tests were inconclusive. We further quantified climatic niche changes using four (for *H. erythraea*) to five (for *R. marina, H. rugulosus*, and *K. pulchra*) PCs to explore alternative PC biplots (see Supplementary Figs. S6–9 and Tables S2).

### Dissimilar multidimensional hypervolumes of realized climatic niches

**Figure 2:**
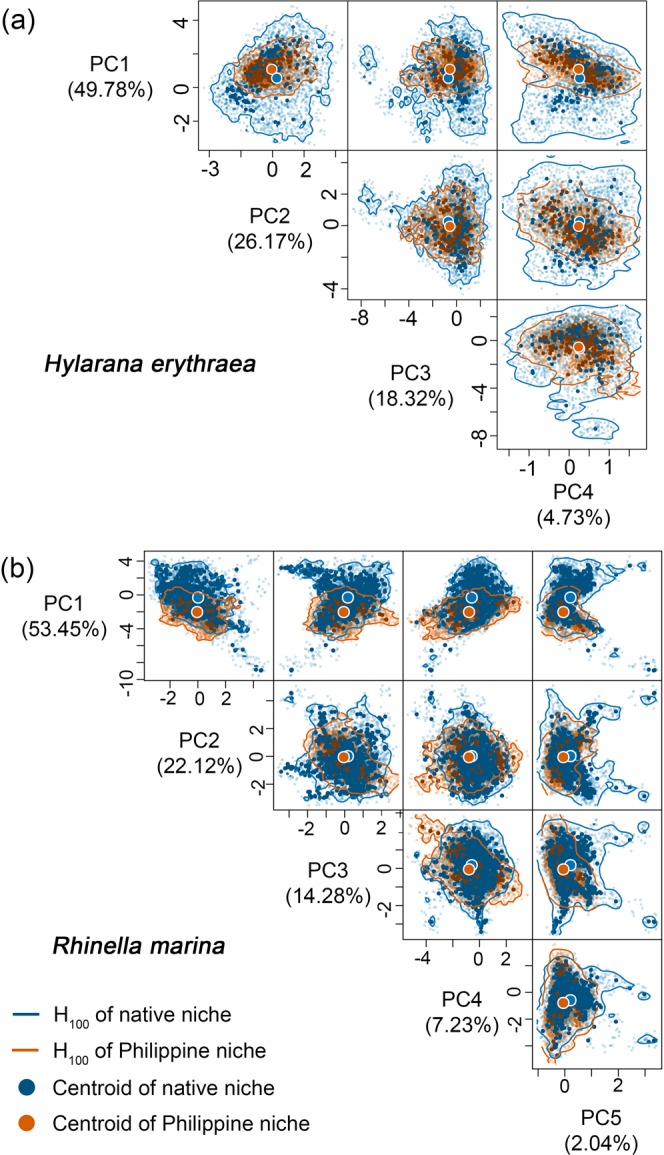
The hypervolumes of the native niche (blue) and Philippine niche (red) of *Hylarana erythraea* (**a**) and *Rhinella marina* (**b**) in multidimensional environmental space defined by four (for *H. erythraea*) to five (for *R. marina*) PCs. The solid contour lines represent the entire boundary of hypervolumes(H_100_). The filled circles represent the centroids of the hypervolumes of the native niche (blue) and Philippine niche (red). Opaque dots represent true species’ occurrence records, whereas transparent dots represent random records derived from Gaussian kernel density estimation.

**Figure 3:**
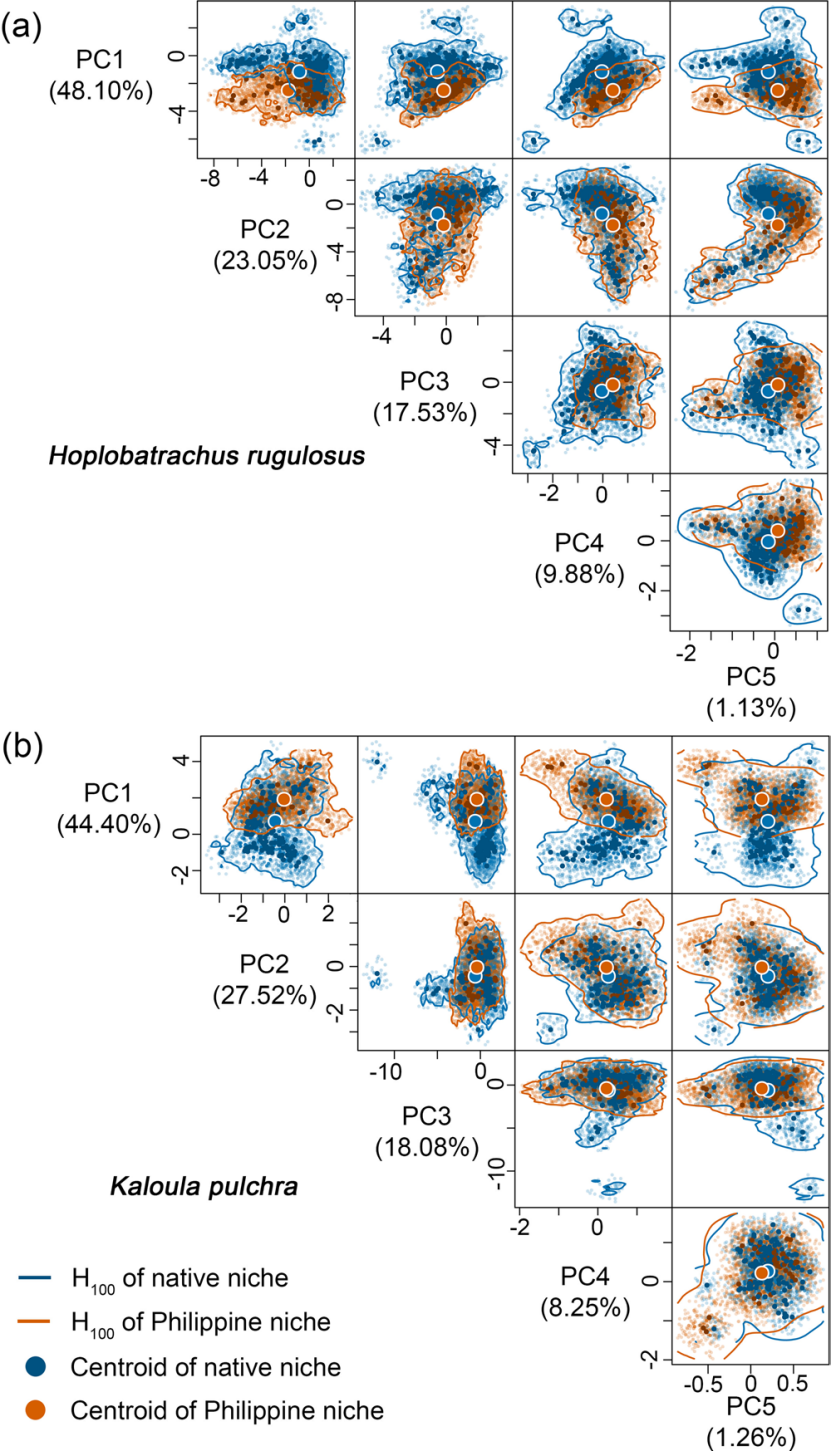
The hypervolumes of the native niche (blue) and Philippine niche (red) of *Hoplobatrachus rugulosus* (**a**) and *Kaloula pulchra* (**b**) in multidimensional environmental space defined by five PCs. The solid contour lines represent the 100% percentile probability boundary of hypervolumes(H_100_). The filled circles represent the centroids of the hypervolumes of the native niche (blue) and Philippine niche (red). Opaque dots represent true species’ occurrence records, whereas transparent dots represent random records derived from Gaussian kernel density estimation.

We compared species’ native and Philippine niches using the *n-*dimensional hypervolume framework^49,50^. Here, we quantified the native and Philippine niches using multidimensional hypervolumes, derived from four (for *H. erythraea*) to five (for *R. marina, H. rugulosus*, and *K. pulchra*) PCs used in the COUE framework. Examining the topology and geometry of the hypervolumes of the native and Philippine niches in multidimensional environmental space revealed: low similarity at both the 75^th^ percentile probability boundary of hypervolumes (H_75_; Jaccard Similarity Index *J* = 0.04–0.18) and entire probability boundary of hypervolumes (H_100_; *J* = 0.11–0.24); low to moderate intersection at both H_75_ (4.73–18.38%) and H_100_ (11.22–22.37%); varying levels of unique fractions of the Philippine niche at H_75_ (15–81%) and H_100_ (8–57%); short centroid distances in *H. erythraea* at H_75_ (0.24) and H_100_ (0.62) relative to the other species (1.23–1.66 at H_75_; 1.30–1.80 at H_100_); and short minimum distances between hypervolumes at H_75_ (0.05–0.15) and H_100_ (0.11–0.17) (Figs. 2–3; Table 2; see Supplementary Figs. S10–13).

**Table 2:** Summary results of hypervolume similarity index and hypervolume distance and intersection metrics.

	***Hylarana erythraea***		***Rhinella marina***		***Hoplobatrachus rugulosus***		***Kaloula pulchra***	
	*H*_*75*_	*H*_*100*_	*H*_*75*_	*H*_*100*_	*H*_*75*_	*H*_*100*_	*H*_*75*_	*H*_*100*_
**Similarity Index** (*Jaccard*)	0.15	0.14	0.18	0.24	0.05	0.11	0.18	0.22
**Hypervolume distance metrics**								
Centroid	0.24	0.62	1.66	1.70	1.60	1.80	1.28	1.30
Minimum	0.05	0.13	0.13	0.11	0.15	0.17	0.15	0.17
**Hypervolume intersection metrics**								
Volume								
*Native niche*	64.10	343.64	69.75	340.60	73.14	351.44	18.63	82.81
*PH niche*	11.30	54.21	22.42	106.42	22.06	106.71	13.36	57.53
*Union*	65.81	348.20	77.86	370.87	90.90	411.93	27.15	114.68
*Intersection*	9.60	49.65	14.31	76.15	4.30	46.21	4.85	25.66
Unique Fraction of the native niche (%)	85	86	79	78	94	87	74	69
Unique fraction of the Philippine niche (%)	15	8	36	28	81	57	64	55

### Undermined performance of native and Philippine EMs

**Figure 4:**
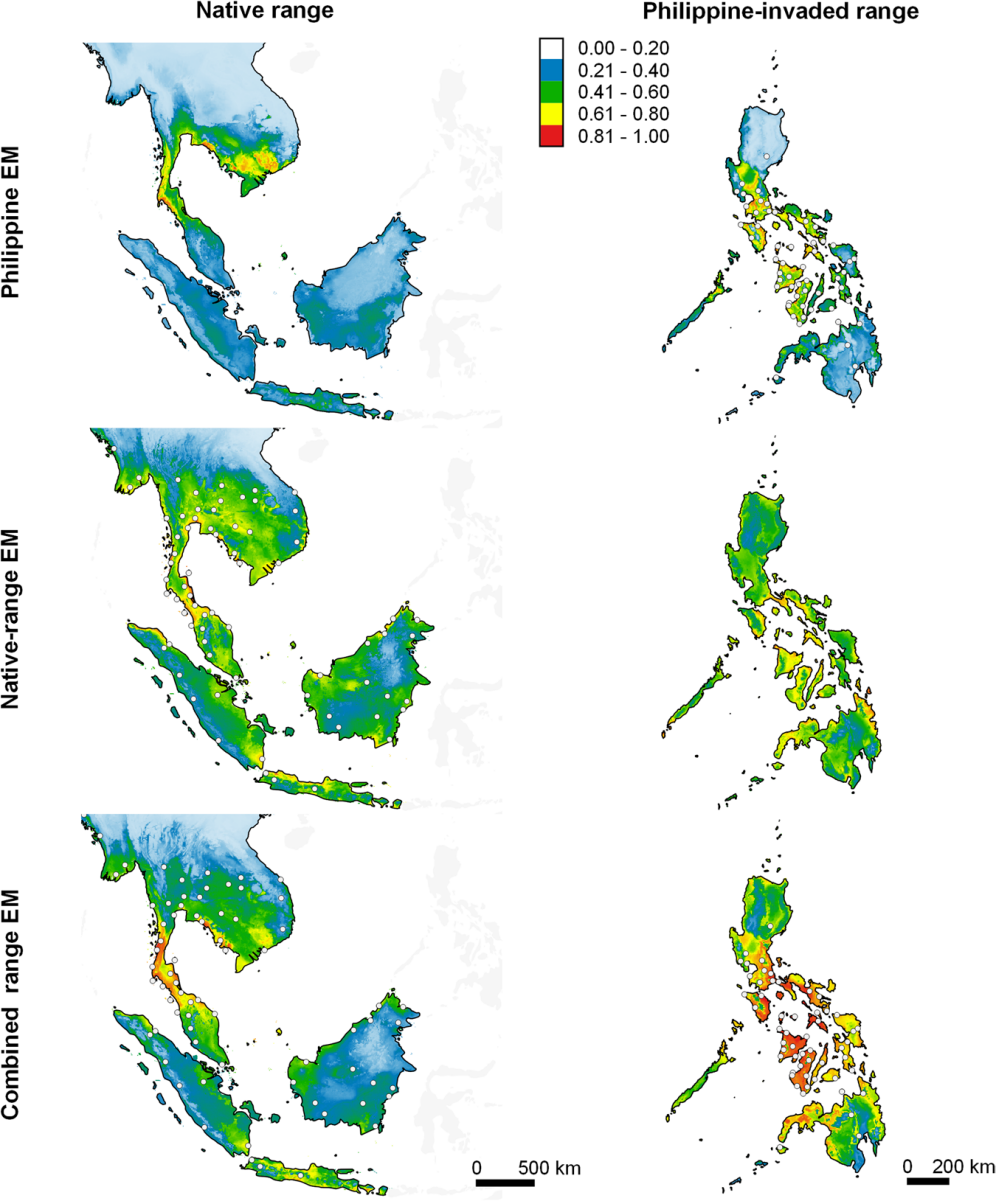
Predicted potential distribution of *Hylarana erythraea* in its native range and in the Philippines (columns) projected by EMs calibrated using data from the Philippine-invaded range, native range, and combined ranges (rows). *Hylarana erythraea* is native to South Asia, mainland Southeast Asia, and parts of maritime Southeast Asia (Borneo, Indonesia [Sumatra, Java, Lombok, and Riau Islands]). White dots represent species occurrence records, which were thinned to improve visibility. Relative climatic suitability increases from cold to warm colours. The maps were created using QGIS Geographic Information System software (v. 3.14; http://qgis.osgeo.org) and projected using WGS 1984 Coordinate Reference System.

**Figure 5:**
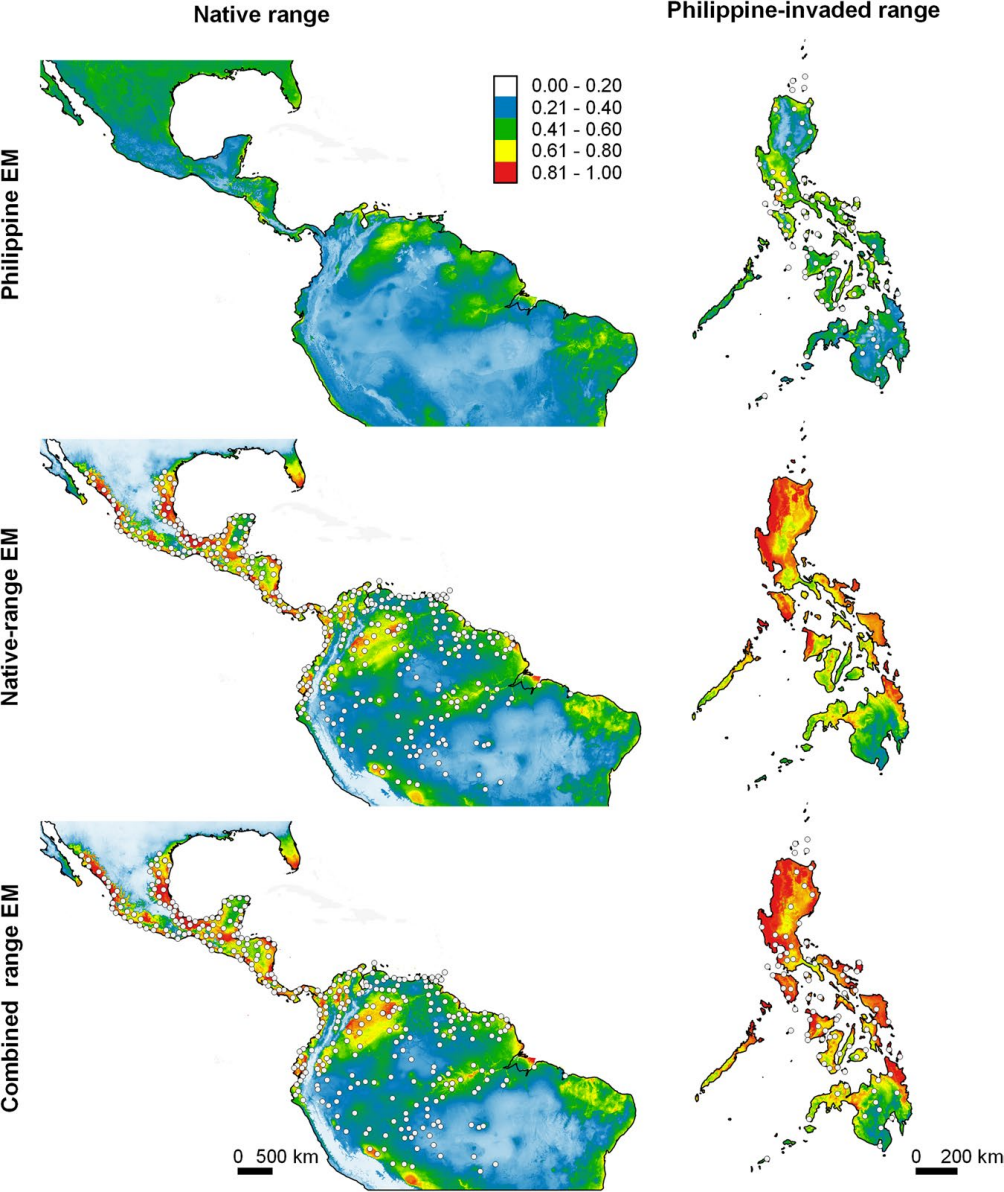
Predicted potential distribution of *Rhinella marina* in its native range and in the Philippines (columns) projected by EMs calibrated using data from the Philippine-invaded range, native range, and combined ranges (rows). *Rhinella marina* is native to tropical Americas. White dots represent species occurrence records, which were thinned to improve visibility. Relative climatic suitability increases from cold to warm colours. The maps were created using QGIS Geographic Information System software (v. 3.14; http://qgis.osgeo.org) and projected using WGS 1984 Coordinate Reference System.

**Figure 6:**
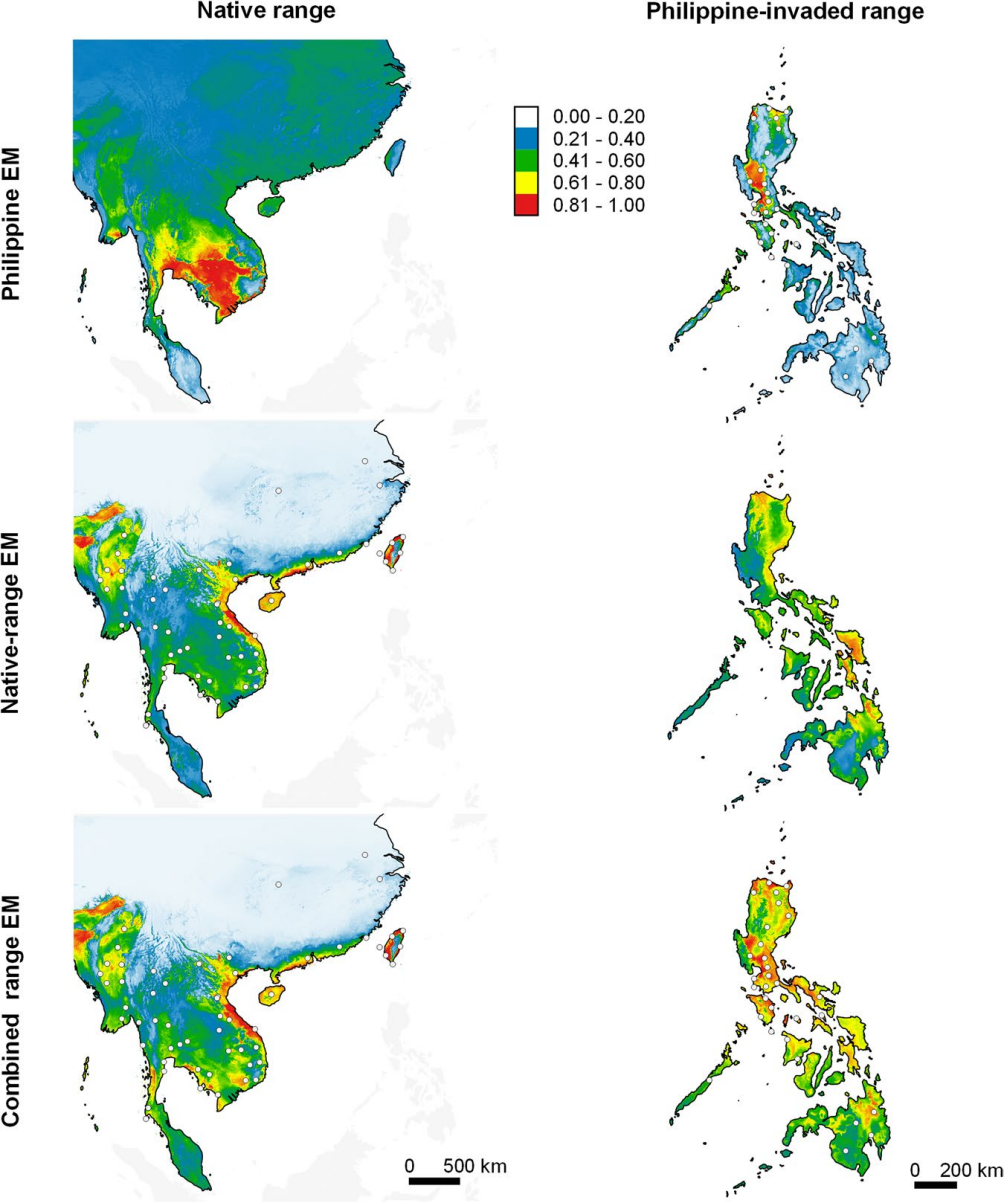
Predicted potential distribution of *Hoplobatrachus rugulosus* in its native range and in the Philippines (columns) projected by EMs calibrated using data from the Philippine-invaded range, native range, and combined ranges (rows). *Hoplobatrachus rugulosus* is native to East Asia (China and Taiwan) and mainland Southeast Asia. White dots represent species occurrence records, which were thinned to improve visibility. Relative climatic suitability increases from cold to warm colours. The maps were created using QGIS Geographic Information System software (v. 3.14; http://qgis.osgeo.org) and projected using WGS 1984 Coordinate Reference System.

**Figure 7:**
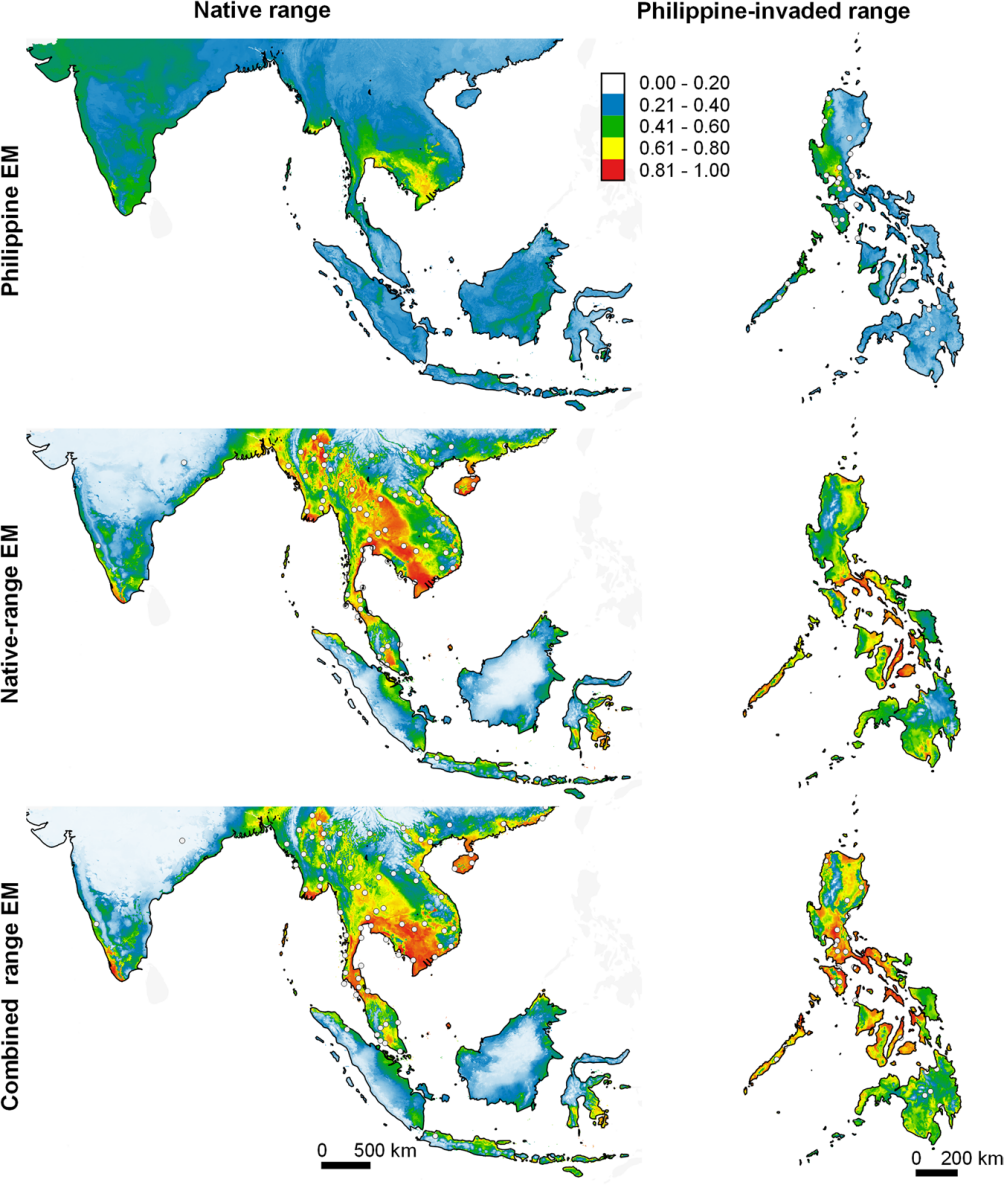
Predicted potential distribution for *Kaloula pulchra* in their native range and in the Philippines (columns) projected by EMs calibrated using data from the Philippine-invaded range, native range, and combined ranges (rows). *Kaloula pulchra* is native to Southern East Asia (China), eastern South Asia (Bangladesh and India), mainland Southeast Asia, and some parts of maritime Southeast Asia (Singapore and Indonesia [Sulawesi]). White dots represent species occurrence records, which were thinned to improve visibility. Relative climatic suitability increases from cold to warm colours. The maps were created using QGIS Geographic Information System software (v. 3.14; http://qgis.osgeo.org) and projected using WGS 1984 Coordinate Reference System.

Ensemble models of Philippine ENMs (Philippine EMs) predicted high climatic suitability in many areas in the Philippines where the species are known to occur (Figs. 4–7). This was supported by the scores of the Area Under the Receiver Operating Characteristic Curve (AUC) and by True Skill Statistics (TSS) derived from projecting Philippine EMs to the evaluation dataset (E_eval_; see methods), wherein AUC scores were fair (*H. erythraea*, *H. rugulosus*) to good (*R. marina*, *K. pulchra*), and TSS scores were consistently high (>0.50) (Table 3; see Supplementary Figs. S14–17). In contrast, the Philippine EMs of all species underpredicted climatically suitable areas in the native range (Figs. 4–7). The AUC of the Philippine EMs, when projected to the species’ presence/pseudo-absence dataset in the native range (E_nat_), ranged from failed (*R. marina* and *H. rugulosus*) to poor (*H. erythraea* and *K. pulchra*), whereas TSS scores were slightly better than random (Table 3; see Supplementary Figs. S14–17). Projecting values of environmental variables in the Philippines to those in species’ native ranges revealed vast extents of clamping of several environmental variables – predictions in these areas are therefore uncertain (see Supplementary Figs. S18–21).

**Table 3:** Evaluation of EMs and their constituent ENMs (columns) of *Hylarana erythraea*, *Rhinella marina*, *Hoplobatrachus rugulosus*, and *Kaloula pulchra* when projected to the 30% evaluation data (E_eval_), Philippine-invaded range (E_inv_), and native range (E_nat_) (rows).

	Philippine ENMs	Philippine EM	Native ENMs	Native EM	Combine range ENMs	Combined range EM
**(a) Area Under the Receiver Operating Characteristic Curve (AUC)**						
***Hylarana erythraea***						
E_eval_	0.75 (0.60–0.80)	0.72	0.69 (0.57–0.79)	0.70	0.78 (0.58–0.82)	0.81
E_inv_	—	—	0.59 (0.48–0.70)	0.65	0.75 (0.61–0.86)	0.81
E_nat_	0.63 (0.47–0.72)	0.61	—	—	0.72 (0.57–0.85)	0.80
***Rhinella marina***						
E_eval_	0.65 (0.50–0.71)	0.87	0.83 (0.78–0.89)	0.86	0.82 (0.78–0.88)	0.85
E_inv_	—	—	0.55 (0.48–0.59)	0.56	0.58 (0.48–0.71)	0.60
E_nat_	0.56 (0.44–0.65)	0.59	—	—	0.84 (0.80–0.93)	0.88
***Hoplobatrachus rugulosus***						
E_eval_	0.74 (0.58–0.81)	0.79	0.88 (0.54–0.93)	0.93	0.92 (0.80–0.96)	0.95
E_inv_	—	—	0.48 (0.30–0.72)	0.44	0.68 (0.41–0.86)	0.82
E_nat_	0.52 (0.29–0.77)	0.55	—	—	0.93 (0.81–0.98)	0.96
***Kaloula pulchra***						
E_eval_	0.69 (0.47–0.8)	0.80	0.85 (0.74–0.91)	0.90	0.85 (0.68–0.88)	0.88
E_inv_	—	—	0.56 (0.36–0.76)	0.59	0.68 (0.45–0.90)	0.77
E_nat_	0.56 (0.39–0.77)	0.61	—	—	0.88 (0.79–0.94)	0.93
**(b) True Skill Statistics (TSS)**						
***Hylarana erythraea***						
E_eval_	0.40 (0.26–0.55)	0.66*	0.32 (0.18–0.50)	0.62*	0.42 (0.22–0.54)	0.65*
E_inv_	—	—	0.10 (–0.02–0.28)	0.20	0.35 (0.16–0.56)	0.30
E_nat_	0.13 (0.04–0.22)	0.09	—	—	0.21 (0.02–0.41)	0.34
***Rhinella marina***						
E_eval_	0.20 (−0.01–0.34)	0.54*	0.51 (0.40–0.63)	0.67*	0.51 (0.44–0.63)	0.63*
E_inv_	—	—	0.04 (−0.07–0.11)	0.05	0.05 (0.00–0.11)	0.08
E_nat_	0.06 (−0.09–0.18)	0.02	—	—	0.54 (0.46–0.71)	0.60
***Hoplobatrachus rugulosus***						
E_eval_	0.39 (0.17–0.53)	0.77*	0.61 (0.08–0.72)	0.84*	0.68 (0.58–0.76)	0.79*
E_inv_	—	—	−0.04 (−0.23–0.42)	−0.10	0.17 (0.00–0.50)	0.31
E_nat_	0.00 (−0.40–0.37)	0.01	—	—	0.72 (0.62–0.87)	0.79
***Kaloula pulchra***						
E_eval_	0.30 (−0.06–0.52)	0.82*	0.61 (0.41–0.70)	0.81*	0.55 (0.43–0.61)	0.79*
E_inv_	—	—	0.10 (−0.27–0.4)	0.01	0.12 (0.00–0.28)	0.26
E_nat_	0.07 (−0.18–0.24)	0.16	—	—	0.64 (0.48–0.72)	0.72

Performance of ENMs and EMs was evaluated using the Area Under the Receiver Operating Characteristic Curve (AUC) (**a**) and True Skill Statistics (TSS) (**b**). Since ENMs were weighted based on their TSS scores when projected to E_eval_, the TSS scores of the resulting EMs when projected to E_eval_ will be equivalent to zero. Here, the TSS scores of EMs when projected to testing data are shown instead (*).

Ensemble models of native ENMs (native EMs) predicted low climatic suitability in many areas in the Philippines where the species are known to occur (Figs. 4–7). Projecting native EMs to the species’ presence/pseudo-absence dataset in the Philippine-invaded range (E_inv_) produced AUC scores ranging from no better than random (*H. rugulosus*), failed (*R. marina* and *K. pulchra*), to poor (*H. erythraea*), whereas TSS scores ranged from no better than random (*H. rugulosus*) to slightly better than random (Table 3; see Supplementary Figs. S14–17). Interestingly, native EMs predicted high climatic suitability in extensive areas in the Philippines that the species have not yet invaded. In contrast, native EMs produced good predictions of the species’ native range (Figs. 4–7). When projected to its E_eval_ dataset, the native EMs of all species scored fair (*H. erythraea*), good (*R. marina*), to excellent (*H. rugulosus*, *K. pulchra*) AUC values, and high TSS scores (Table 3; see Supplementary Figs. S14–17). Projecting values of environmental variables in the species’ native ranges to those in the Philippines revealed vast extents of clamping of several environmental variables (see Supplementary Figs. S18–21).

When projected to the Philippines and the native range, EMs of combined range ENMs (combined range EMs) predicted high climatic suitability in many areas where the species are known to occur, as well as in areas in the Philippines where the species have not yet invaded (Figs. 4–7). When projected to E_inv_ and E_nat_, the combined-range EMs of all species had fair to excellent AUCs. Meanwhile, the combined-range EMs of most species had lower TSS when projected to E_inv_ and E_nat_ relative to E_eval_ (Table 3; see Supplementary Figs. S14–17). Projecting values of environmental variables in the species’ combined ranges to either their native range or the Philippines revealed negligible clamping of environmental variables.

## Discussion

Our findings revealed varying levels of niche change across four alien amphibian species introduced to the Philippines: high niche stability and low to moderate niche unfilling across all species, and insignificant to substantial niche expansion in all species except *H. erythraea*. All species except *H. erythraea* occupied hotter and wetter climates in the Philippines. Niche overlap was low for all species. Despite the low overlap, niche equivalency tests revealed niche conservatism in *R. marina* and *K. pulchra*, and niche similarity tests revealed niche conservatism in *K. pulchra* and to a lesser extent in *R. marina* and *H. erythraea*. The native and invaded niches of *H*. *rugulosus* were not equivalent, although niche similarity tests were inconclusive. Nonetheless, niche expansion in analogous (i.e., climates found in both the Philippines and each species’ native range) and non-analogous environmental space provide evidence of niche shift in *H. rugulosus*. Niche unfilling revealed environmental non-equilibrium in the Philippine-invaded range for all species, whereas niche expansion revealed environmental non-equilibrium in the native range and/or adaptation in the invaded range for all species except *H. erythraea*. Hypervolume centroid distances consistently supported a shift in the niche centroids of all species except *H. erythraea*. Meanwhile, hypervolume intersection metrics generally revealed substantial unique fractions of Philippine niches, which is overall inconsistent with the findings of the COUE niche change metrics. Consistent with the findings of the niche overlap tests based on Schoener’s *D*, values of Jaccard hypervolume similarity index were consistently low for all species. Finally, we found that native and Philippine EMs showed poor reciprocal transferability – that is, climatic characteristics of the Philippine niche poorly predicted the potential distribution of the species in the native range, and vice versa. Combined range EMs produced relatively better predictions of potential distributions across both native and invaded ranges.

Our findings build on previous studies^32,33^, contributing to our growing understanding of climatic niche changes during the invasion of alien species, in general, and alien amphibian species, in particular. Our study is the first to assess niche changes in *H. erythraea, H. rugulosus*, and *K. pulchra*. Meanwhile, our findings of niche conservatism in the Philippine-invaded range of *R. marina* is somewhat consistent with the findings of Li *et al*.^32^ – that is, the realized climatic niche of *R. marina* is conserved in its Indomalayan invaded range (including the Philippines); our findings showed niche equivalency and to a lesser extent similarity in *R. marina* in its Philippine-invaded range (Table 1), whereas Li *et al*.^32^ showed niche similarity (*P* = 0.02; niche equivalency test was not conducted) in its Indomalayan invaded range. The inconsistent results of the niche similarity test between our study and Li *et al*.^32^ were likely driven by the differences in the scale and geographic extent of our analysis (Philippines only vs. Indomalayan realm, respectively). Our study also extends the work of Tingley *et al*.^33^, who examined niche shifts across the native vs. Australian-invaded range of *R. marina*.

The observed niche shifts could either be due to shifts in the species realized climatic niches in their invaded ranges^27^, to a shift in the species’ fundamental niches (e.g., changes in their environmental tolerances) during the course of invasion^26,37^, or both. Realized climatic niche shift is in line with the notion that species occupy a subset of their fundamental niche in their native range – indicating environmental non-equilibrium – due to dispersal limitations and/or biotic interactions^59,60^. In contrast, fundamental niche shift may be caused by adaptive changes post-introduction^37^. In the case of *R. marina*, we detected expansion of the species’ Philippine niche into analogous and non-analogous environments. Phenotypic changes have been observed over the course of the species’ invasion in Australia^61,62^. However, Tingley *et al*.^33^, by combining ecophysiological and correlative models, found that *R. marina* fails to occupy a substantial fraction of its fundamental niche in its native South American range, plausibly due to biotic interactions with closely related species. More importantly, they showed that *R. marina* has successfully filled its fundamental niche in its Australian-invaded range, reflecting realized climatic niche shift. In our analysis, the captured environmental backgrounds in the native range (derived from biomes intersecting the species’ range) are likely more environmentally restricted than the fundamental niche, and the detected non-analogous climates in the Philippines are likely within the species’ fundamental niche. Meanwhile, for *H. rugulosus*, discriminating whether realized or fundamental niche shift explains the observed niche expansion in non-analogous environmental space would require mechanistic approaches based on experimental measurements of the species’ fundamental niche^26,33,63^.

We extended our main analysis by quantifying niche changes in the COUE framework^47^ using alternative biplots constructed from combinations of the first four to five PCs. Interestingly, alternative biplots revealed inconsistent support for niche equivalency and similarity, and in some cases, high variance in niche change metrics (see Supplementary Table S2). Nonetheless, we argue that the biplots of the first two PCs, as presented here, provide the most reliable results, since they explain the highest amount of variation in the environmental data (>71%). The fact that the COUE framework is limited to analysing niches in two dimensions at a time is a significant limitation. To overcome this limitation, we further quantified species’ climatic niche changes using the *n-*dimensional hypervolume framework^49,50^. Using four to five PCs that collectively captured ~99% variance in the environmental data, our findings again revealed varying levels of niche changes among species, which were generally inconsistent with our findings derived using COUE framework’s niche change metrics. This highlights the difficulty of quantifying niche changes, as well as testing for niche conservatism and shift, due to varied conceptual theories underpinning different methodological frameworks. Nonetheless, the *n-*dimensional hypervolume framework, as currently implemented, does not account for environmental availability in each range (and thus analogous and non-analogous environments) and does not give greater weight to PCs that explain more environmental variation. To this end, we base our conclusions regarding niche conservatism and environmental equilibrium primarily from the findings revealed by the COUE framework.

Should we anticipate future niche changes in the four alien amphibian species’ Philippine niches? We found niche unfilling for all species, revealing environmental non-equilibrium in the species’ Philippine niches. This supports the findings of Pili *et al*.^52^ that all species’ invasions in the Philippines are incomplete, and that the species continue to spread. Moreover, for all species, PC biplots revealed analogous environmental space that is unoccupied in both native and Philippine-invaded ranges. If accessible (in geographic space) and climatically suitable, these areas of environmental space present opportunities for future niche expansion. For *H. rugulosus*, PC biplots revealed unoccupied non-analogous environmental space in the Philippines, which presents opportunities for the species to further shift its Philippine niche. This emphasizes the need for regular reassessments of niche conservatism/shift as a species’ invasion progresses.

Our findings do not conform to observed patterns linking niche unfilling with aspects of species’ invasion history. Patterns in alien amphibian^32^, reptile^32^, and bird^31^ invasions showed that residency time (i.e., the length of time since a species was first introduced) is inversely correlated with the magnitude of niche unfilling. Our findings regarding *R. marina*, which was introduced in the Philippines in 1934^52^, are somewhat conforming with this pattern. Meanwhile, the other species showed an inverse pattern; for example, *H. rugulosus* and *K. pulchra*, respectively introduced only in the 1990s and 2000s^52^, showed low levels of niche unfilling, whereas *H. erythraea* showed substantial niche unfilling, despite being first introduced in the Philippines for more than a century ago in the 1880s^52^.

Niche unfilling in birds^31^ and in alien species in disjunct geographic areas (e.g., archipelagic systems)^33^ have been linked to dispersal limitations (e.g., biogeographic barriers)^64^ and propagule pressure (i.e., the absolute number of individuals involved in a human-mediated dispersal event and the number of discrete dispersal events)^59^. Similarly, the high niche unfilling in *H. erythraea* observed here may be due to its dependence on pathways characterized by low inter-island propagule pressure, especially during the early periods of its invasion. *Hylarana erythraea* is known to historically disperse inter-island primarily as a contaminant of commodities (agriculture, animals) (*sensu* Scalera *et al*.^65^)^52^. The trade of these commodities (reflecting propagule pressure) is high intra-islands, especially on larger Philippine islands such as Luzon and Mindanao, where rapid and short-distance transport is possible and is critical for such perishable commodities. However, inter-island trade of these commodities is low, likely limiting the species dispersal throughout the archipelago. This low level of propagule pressure coincides with its spatio-temporal spread in the Philippines, in which it was first introduced and restricted among import-dependent small islands in the Central Philippines for more than a century, and only dispersed to the country’s major islands of Luzon and Mindanao in the 1990s, where it underwent an increased rate of spread^52^. Meanwhile, high propagule pressure was likely an important contributor to the low levels of niche unfilling in *R. marina*, *H. rugulosus*, and *K. pulchra*. *Rhinella marina* was initially intentionally dispersed in many parts of the Philippines as a biological control agent, where it subsequently dispersed intra-islands primarily via natural dispersal. Moreover, the rapid inter-island dispersal of *R. marina* has been attributed to the Philippines’ dependence on sea transport for trade and travel – this species has been observed to hitchhike on transportation vehicles (e.g., ships, boats, trucks)^52^. The use of multiple alternative introduction and dispersal pathways – providing alternative ways for intra- and inter-island dispersal – likely aided *H. rugulosus* and *K. pulchra* in negating effects of residency time and dispersal limitations, resulting in rapid dispersal throughout the country^52^.

Predictions of native EMs corroborated previous studies showing that ENMs based on the native range of species often underpredict suitable areas in the invaded range^26,29,40–42^. Specifically, native EMs predicted low climatic suitability in many areas in the Philippines where the species occur. The observed poor predictive performance of native EMs was likely caused by the observed niche expansion and unfilling in the species Philippine niches. Interestingly, our findings, especially for *H. erythraea*, support previous studies^31,35^ showing increased predictive performance of native ENMs with decreasing niche unfilling and expansion in the invaded range. Meanwhile, Philippine EMs support previous studies showing ENMs calibrated using invaded range data may be able to capture niche shifts that occurred during invasion^26,29,40–42^. However, due to environmental non-equilibrium often observed in alien species in their invaded ranges, ENMs calibrated using invaded range data may fail to capture parts of the species’ climatic niche that is occupied in the native range but unoccupied in the invaded range. This leads to underprediction of the species’ potential distribution in both the native and invaded ranges^22,23,25,41^, as observed in the predictions of the Philippine EMs. Overall, combined range EMs predicted high climatic suitability in areas in the Philippines where the species are known to occur, and predicted areas that are climatically suitable but are yet uninvaded by the species. Our findings corroborate those of previous studies in that combining data from the native and invaded ranges of an alien species, where available, can offset the predictive limitations of ENMs calibrated in only the native or invaded ranges^26,29,40–42^. We recommend future researchers recalibrate combined range EMs as species’ invasions progress, and when data from other ranges of the species are collected and/or made available.

## Conclusion

Overall, our findings revealed evidence of conservatism in the Philippine niches of *R. marina* and *K. pulchra*, and a shift in the Philippine niche of *H. rugulosus*. Meanwhile, for *H. erythraea*, the extent to which the species’ niche has been conserved is less clear. Different aspects of species’ invasion history, such as residency time, dispersal limitation, and propagule pressure, can only partially explain the observed changes in the species’ Philippine niches. The observed niche changes, reflecting niche conservatism/shifts and environmental non-equilibrium, likely caused the observed poor reciprocal transferability of native and Philippine EMs and their constituent ENMs. Our findings support previous studies showing that ENMs calibrated with data from the combined native and invaded ranges, where available, will be most useful in informing invasion risk assessments^22,23,35,41,66^. In light of the implications of niche shifts and environmental non-equilibrium for ENM predictions, we suggest researchers and managers routinely incorporate quantification of niche changes into ecological niche modelling experiments^23,27,42,47,48^. In addition, the fact that the majority of alien species’ invasions are incomplete^23,25,41,43^, such as the case of the four alien amphibian species studied here, emphasizes the need for researchers and managers to regularly reassess niche changes in alien species’ invaded ranges as a species’ invasion progress^33^. Consequentially, ENMs must be regularly recalibrated with updated data to reflect concurrent changes in the species’ realized climatic niche in the invaded range^33,40,58^.

## Materials and Methods

### Species occurrence and environmental data

Occurrence records of the four alien amphibian species in the Philippines and the species’ native ranges were obtained from the Global Biodiversity Information Facility (GBIF)^67^, collections of local natural history institutions, scientific publications, expert observations, and field surveys conducted by the authors (see Supplementary Methods)^57^. The pooled dataset was cleaned manually so that only high-quality records were used in the analysis; records conforming to these set of conditions were retained: (1) georeferenced; (2) with year of record; (3) with “county” or “municipality” locality data (*sensu* Darwin Core Task Group^68^); (4) inland coordinates and within the ascribed locality (up to the third administrative level [admin2] of the Global Administrative Areas v3.6 [https://gadm.org/]) – assessed in InfoXY v.2 tool of speciesLink (http://splink.cria.org.br/); (5) and inside the known native range of the species, as depicted by species range maps^69–72^. To minimize spatial sampling bias, occurrence records were thinned by subsampling records to a resolution of one record per 5 km^2^ (2.5 arc-min)^73,74^. Initial visual assessment of the spatial distribution of the records of *R. marina* showed sampling bias across its native range, where records were denser in Central America relative to other parts of its range. Thus, we further reduced native-range records of *R. marina* to a resolution of 10 km^2^ (5 arc-min)^33^. We initially planned to omit occurrence data outside the years 1970–2000, to align with the temporal reference of the environmental variables (described below)^75^. However, this would have resulted in the omission of a considerable proportion of collected occurrence data (~60–100%), especially data on recent range expansions of the species in the Philippines (*K. pulchra* was first recorded in the 2000s), and would inevitably negatively affect our analysis of realized climatic niche changes and our modelling of species’ ecological niches. In addition, after our extensive data cleaning process, the final thinned dataset only contained data recorded from 1950 to present (Table 4).

**Table 4:** Native and Philippine-invaded range occurrence records used to calibrate the ecological niche models.

	Native range	Philippine-invaded range	Combined ranges
*Hylarana erythraea*	143	152	295
*Rhinella marina*	1,581	170	1,751
*Hoplobatrachus rugulosus*	248	94	342
*Kaloula pulchra*	164	48	212

### Environmental variables

Extreme temperature and precipitation can negatively affect amphibian development (e.g., tadpole and egg developmental rates/morbidity), ecophysiology (e.g., locomotor performance, communication, sensory systems), and energy acquisition and allocation^76–78^. Temperature and precipitation seasonality alter growth, reproductive cycles, phenology, and prey-predator dynamics^79–82^. At a broad geographical scale, precipitation and temperature extremes and seasonality influence amphibian biogeography (population declines and extirpations, shifts in geographic distribution, species extinction)^83–85^. Thus, in quantifying realized climatic niche changes and ecological niche modelling, we used environmental variables representing a combination of means, extremes, and seasonality that are known to be ecologically relevant to amphibians and are not highly inter-correlated (Pearson’s correlation coefficient |r | ≤ 0.7)^86^: annual mean temperature (bio 1), temperature seasonality (bio 4), maximum temperature of the warmest month (bio 5), minimum temperature of the coldest month (bio 6), annual precipitation (bio 12), precipitation seasonality (bio 15), precipitation of the wettest quarter (bio 16), and precipitation of the driest quarter (bio 17). Bioclimatic variables were obtained from WorldClim v.2, and represent averages of monthly minimum, mean, and maximum temperature and of precipitation for 1970-2000^75^ with a spatial resolution of 5 km^2^ (2.5 arc-minutes).

### Quantifying niche changes

We quantified the native and Philippine niches of the four alien amphibian species using the COUE framework^47^ and the *n*-dimensional hypervolume framework^49,50^. We then tested for niche equivalency and similarity between the native and Philippine niches of the species^48,51^. Finally, we assessed for niche conservatism and environmental equilibrium based on findings of the methodological frameworks and niche equivalency and similarity tests.

### COUE Approach

Using the COUE framework^47^, we quantified the species’ native and Philippine niches in weighted PC biplots and decomposed climatic changes in species’ niche using a unified set of metrics^27,47^. We analyzed this using the *ecospat* package^87^ in R v.3.6^88^. We first transformed each species’ global environmental space based on the eight environmental variables described above, onto a biplot defined by the first two PCs of a PCA. We calibrated the PCA using pooled environmental backgrounds from the species’ native and Philippine ranges. We defined ecologically relevant environmental backgrounds following the suggestions of Guisan *et al*.^47^. In the native range, environmental backgrounds include all the biomes^89^ inhabited by the species (i.e., biomes that are intersected by the species’ native range^69–72^). In the Philippines, environmental backgrounds included the whole of the Philippines. The first two PCs captured ~71–76% of the variation in the environmental data. The correlations of environmental variables with the PCs are shown in Supplementary Table S1 (online). We then divided the species’ global environmental space, as depicted in a PC biplot, into a grid consisting of 100 ×100 cells, bounded by the minimum and maximum values in the environmental background. Lastly, we projected the scores of the species occurrence records onto the gridded environmental space and grouped the scores per grid cell. We applied a Gaussian kernel density function with a standard bandwidth to estimate the smoothed density of occurrences in each cell of the gridded environmental space^90^. We further quantified the species realized climatic niche using alternative biplots from combinations of the first four (for *H. erythraea*) to five (for *R. marina, H. rugulosus, and K. pulchra*) PCs (see Supplementary Figs. S6–9).

We visually examined the niches’ occurrence densities in gridded environmental space, as depicted in a PC biplot, and categorized their topological interaction into five possible patterns^58^: (1) complete to near-complete overlap of the two niches; (2) the Philippine niche is a subset of the native niche; (3) native niche is a subset of the Philippine niche; (4) partial overlap between the two niches; or (5) completely disjunct niches. We decomposed niche changes in analogous environmental space into niche stability, niche expansion, and niche unfilling^27,47^. We calculated these indices in I_75_ (to remove marginal climates resulting from kernel smoothing) and I_100_ of environments available in each range. These indices are not confounded by non-analogous climates, making measured expansion indicative of changes in the realized niche^27^. Thus, we also visually assessed niche unfilling and expansion in non-analogous environmental space.

### Niche overlap, equivalency, and similarity

We quantified the overlap between each species’ native and Philippine niches using Schoener’s index *D* of niche overlap^91^, which estimates the overall similarity between the two niches over the global environmental space^51^. Schoener’s *D* ranges from zero (no overlap) to one (complete overlap)^51,91]^. We quantified niche conservatism by testing estimates of *D* under two alternative hypotheses, representing two extremes across a spectrum of niche conservatism: (1) *niche equivalency* which determines whether realized niches of two entities in two geographical ranges are conserved in the strictest sense (i.e., whether observed niche overlap is effectively indistinguishable when randomly re-allocating pooled occurrences of both niches between them), and (2) *niche similarity*, which estimates whether the realized niche occupied in one range is more similar to the niche occupied in the other range than randomly generated niches^48,51^

For each species, we tested the hypothesis of niche equivalency by simulating two niches based on randomly permuted pooled occurrence records of the native and Philippine niches (the simulated niches maintain the same number of occurrence records as that observed between the native and Philippine niches). The overlap between the two simulated niches was then estimated using Schoener’s *D*. This process was repeated 1,000 times to generate a null distribution of niche overlap values of simulated randomly permuted niches and to confidently reject/accept the hypothesis of niche conservatism. We inferred niche conservatism if the observed niche overlap was greater than 95% of null distribution values (using the “greater” test of *ecospat.niche.equivalency.test* function in the *ecospat* package, which is a one-tailed test). In contrast, observed values outside the density of 95% of null distributions suggest a significant difference in niches^48,51^.

We tested for niche similarity by simulating two niches based on occurrence densities with randomly placed centroids in the environmental background using two randomization tests: (1) centroids randomly placed in both the native and Philippine range (N ↔ P) and (2) centroid randomly placed in the Philippine-invaded range only (N → P)^48,51^. The simulated niches maintain the same number of occurrence records as that observed between the native and Philippine niches. This process was repeated 1,000 times, randomly shifting the centroid of simulated occurrence densities in each repeat, to generate a null distribution of niche overlap values of simulated random niches and to confidently reject/accept the hypothesis of niche conservatism. We inferred niche conservatism if the observed overlap value was greater than 95% of null distribution values (using the “greater” test of *ecospat.niche.similarity.test* function in the *ecospat* package)^48,51^.

### *n*-dimensional hypervolume framework

We analysed the multidimensional hypervolumes of species’ native and Philippine niches using the *hypervolume* package^92^ in R v.3.6^88^. Using the Gaussian kernel density estimation method, we generated multidimensional hypervolumes of species native and Philippine niches from homogeneously distributed random records. These random records were derived from the Gaussian kernel density estimates of the distributions of species’ occurrence records in multidimensional environmental space comprising of the same four (for *H. erythraea*) to five (for *R. marina, H. rugulosus*, and *K. pulchra*) PCs used in the COUE framework, which collectively capture ~99% of variation in environmental data. The bandwidth for Gaussian kernel density estimates was estimated separately for each species niche using the Silverman bandwidth estimator (see Supplementary Table S3)^89^. The hypervolumes of the species’ native and Philippine niches were compared using a similarity index (Jaccard) and niche changes were decomposed using hypervolume distance (minimum distance, centroid distance) and intersection (volume of the intersection, the unique fraction of hypervolumes) metrics^93^. We calculated the hypervolume metrics and similarity index in H_75_ (to remove marginal climates resulting from kernel smoothing) and in H_100_.

### Ecological niche modelling

We modelled the realized climatic niches of the four alien amphibian species using eight statistical approaches: (1) Generalized Additive Model (GAM)^94^, (2) Generalized Linear Model (GLM)^95^, (3) Multivariate Adaptive Regression Splines (MARS)^96^; a classification technique – (4) Classification and Regression Trees (CART)^97^; machine learning techniques –(5) Artificial Neural Networks (ANN)^98^, (6) Random Forests (RF)^99^, (7) Boosted Regression Trees (BRT)^100^, and (8) Maximum Entropy (Maxent)^101^. We calibrated ENMs with the same eight environmental variables used to quantify the realized climatic niche changes and occurrence records from the species’ (1) Philippine-invaded range, (2) native range, and (3) combined ranges. We tested for environmental variable clamping to identify locations where values of variables were outside the range used for calibrating ENMs – predictions in these areas are uncertain. This was done by projecting values of environmental variables from one range to another (e.g., the Philippines to species’ native range). Barbet-Massin *et al*.^102^ laid-out optimal settings in generating and weighting pseudo-absences for different statistical techniques used in ENM. Here, we generated and weighted pseudo-absences to yield good to optimal ENM performance across the eight statistical techniques^102^. We generated pseudo-absence data by randomly sampling 10,000 background points from areas where the species has no presence records and across all the biomes^89^ inhabited by the species (same environmental backgrounds used in COUE framework). For each statistical approach, we equally weighted the presences and pseudo-absences used to calibrate the ENMs by setting a neutral (0.5) prevalence. We conducted the analysis using the *biomod2* package^103,104^ in R v.3.6^88^. We ran all ENMs with the default parameters of the statistical techniques.

Prior to modelling, we randomly split each presence/pseudo-absence dataset (datasets for native, Philippine, and combined range ENMs) into two parts: 70% for training and 30% for evaluation (E_eval_). By assigning a specific subset for evaluation, the EMs (discussed below) and its constituent ENMs were evaluated using the same data, ensuring a fair evaluation between the performances of EMs compared to ENMs^104^. For each species, we further sub-sampled the training data subset randomly to 70% training and 30% testing. This was repeated 10 times to account for uncertainty due to random subset selection, resulting in 10 random sub-samples of training-testing data. We modelled each sub-sample of training-testing data using the eight statistical approaches described above, generating 80 Philippine ENMs, 80 native ENMs, and 80 combined ENMs per species.

To evaluate predictive performance, we projected the ENMs to their respective E_eval_. To evaluate reciprocal transferability, we projected the Philippine ENMs to the entire presence/pseudo-absence dataset in the native range (E_nat_), native ENMs to the entire presence/pseudo-absence dataset in the Philippine-invaded range (E_inv_), and combined ENMs to both E_nat_ and E_inv_. We then computed for the AUC^105^ and the TSS^106^. We interpreted the AUC values based on Swets *et al*.^107^, where values >0.90 = excellent, >0.80–0.90 = good, >0.70–0.80 =fair, >0.60–0.70 = poor, and >0.50–0.60 = fail. Meanwhile, TSS values range from –1 to 1, where 1 indicates perfect agreement and values zero or less indicate performance no better than random^106^.

For each species, we combined each set of ENMs (80 Philippine ENMs; 80 native ENMs; 80 combined ENMs) to build a consensus ensemble model where each ENM was weighted by its TSS scores (i.e., better performing models contribute more to the EM)^56,104^. We then evaluated the EMs using the evaluation datasets used in evaluating their constituent ENMs (E_eval_ and E_nat_ for Philippine EMs; E_eval_ and E_inv_ for native EMs; E_eval_, E_inv_, and E_nat_ for combined range EMs). To predict species’ potential distributions, we projected species’ EMs to the Philippines and their respective native ranges.

## Supplementary information

Supplementary Information.

## Data Availability

All data was gathered from publicly available sources and are available in the online registry of the Global Biodiversity Information Facility (HerpWatch Pilipinas, Inc.;^57^ see Supplementary Methods).
