# Supplementary material for: Niche shifts and environmental non-equilibrium undermine the usefulness of ecological niche models for invasion risk assessments
Source: Sci Rep. 2020 May 14;10:7972. doi: 10.1038/s41598-020-64568-2 (PMC7224218; doi:10.1038/s41598-020-64568-2)
Supplement: Supplementary file 1 — Supplementary Information. [file 41598_2020_64568_MOESM1_ESM.pdf]

Supplementary Information

Niche shifts and environmental non-equilibrium undermine the usefulness of ecological niche models for invasion risk assessments

Arman N. Pili[1,2,3,*], Reid Tingley[3], Emerson Y. Sy[2,4], Mae Lowe L. Diesmos[5,6], Arvin C. Diesmos[1,2,7]

[1]The Graduate School, University of Santo Tomas, España, 1015 Manila, The Philippines;

[2]HerpWatch Pilipinas, Inc., Tondo, Manila, The Philippines

[3] School of Biological Sciences, Monash University, Clayton 3800, Victoria, Australia.

[4] Philippine Center for Terrestrial and Aquatic Research, Tondo, Manila, The Philippines

[5]Department of Biological Sciences, College of Science, University of Santo Tomas, España, 1015. Manila, The Philippines

[6]Research Center for the Natural and Applied Sciences, University of Santo Tomas, España, 1015. Manila, The Philippines

[7]Philippine National Museum of Natural History, T.F. Valencia Circle, T.M. Kalaw Street, Rizal Park, 1000 Manila, Philippines

*Corresponding author:

E-mail: armannorciopili@gmail.com

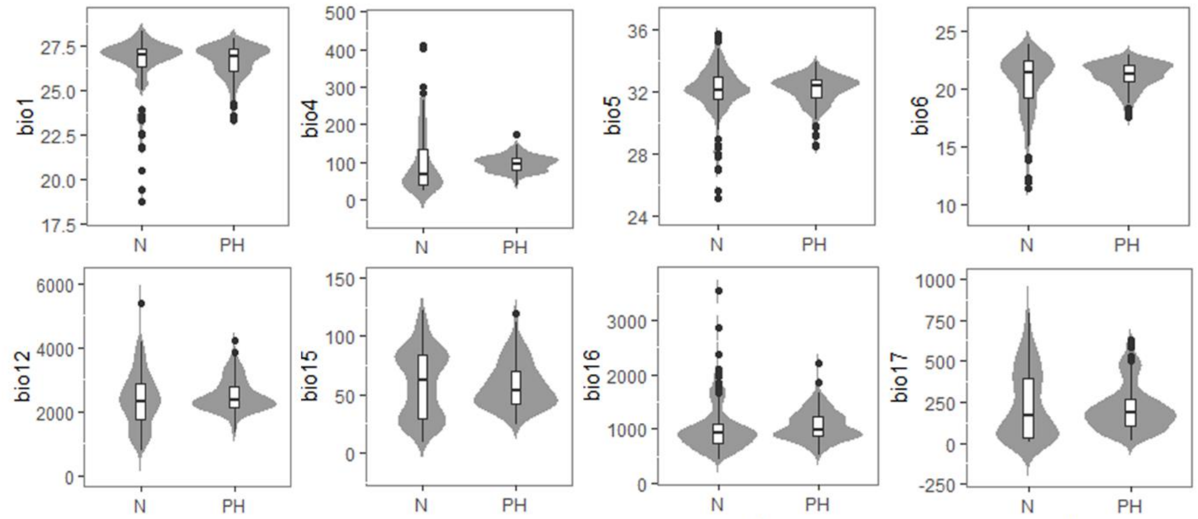

Fig. S1. Environmental variable scores (bar-and-whisker plots) and densities (violin plots) as observed at native (N) and Philippine-invaded range (PH) records of *Hylarana erythraea*. The environmental variables used include: bio1 = annual mean temperature; bio4 = temperature seasonality; bio5 = maximum temperature of warmest month; bio6 = minimum temperature of coldest month; bio12 = annual precipitation; bio15 = precipitation seasonality; bio16 = precipitation of wettest quarter; bio17 = precipitation of driest quarter.

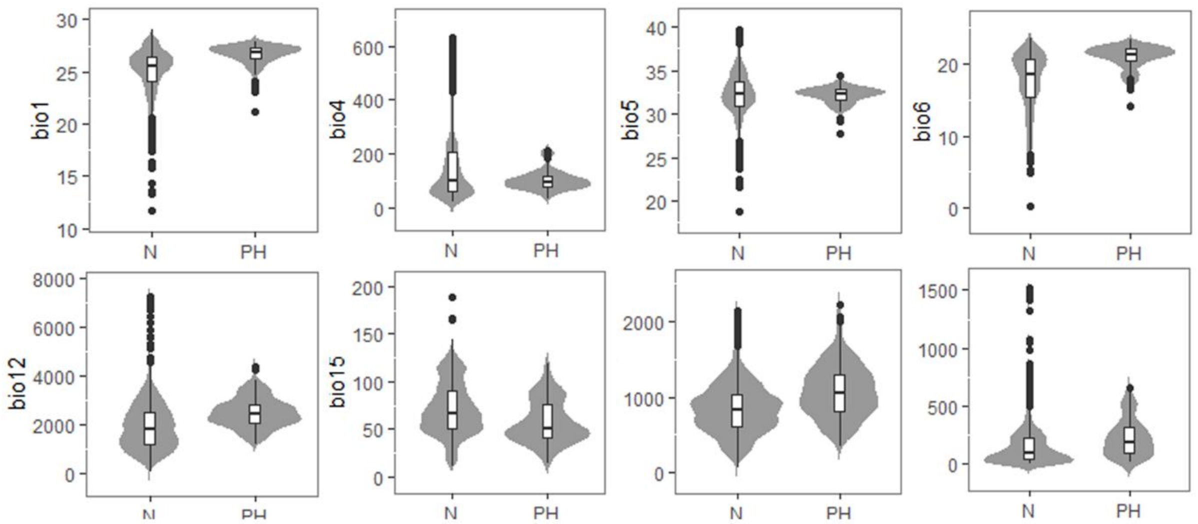

Fig. S2. Environmental variable scores (bar-and-whisker plots) and densities (violin plots) as observed at native (N) and Philippine-invaded range (PH) records of *Rhinella marina*. The environmental variables used include: bio1 = annual mean temperature; bio4 = temperature seasonality; bio5 = maximum temperature of warmest month; bio6 = minimum temperature of coldest month; bio12 = annual precipitation; bio15 = precipitation seasonality; bio16 = precipitation of wettest quarter; bio17 = precipitation of driest quarter.

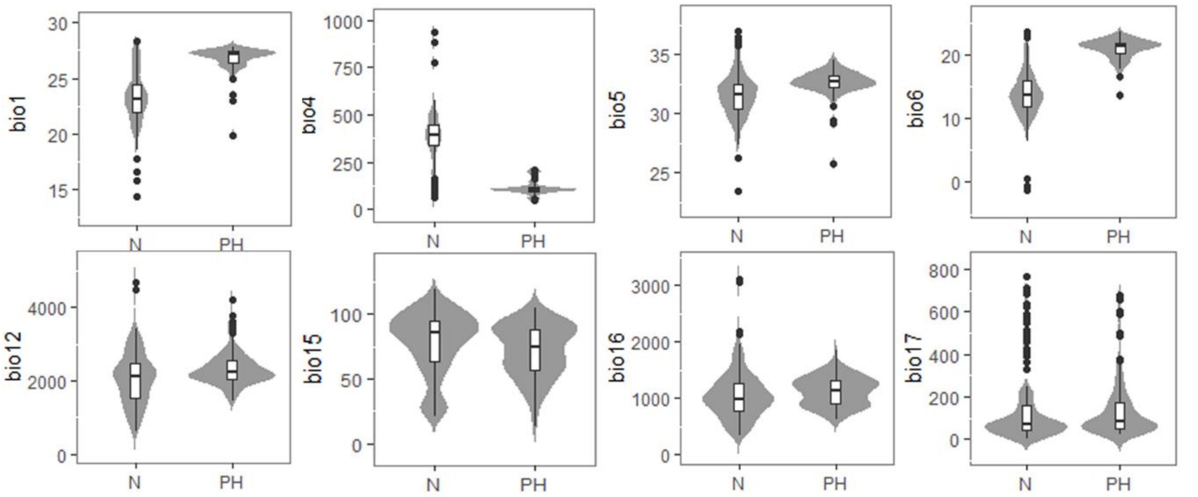

Fig. S3. Environmental variable scores (bar-and-whisker plots) and densities (violin plots) as observed at native (N) and Philippine-invaded range (PH) records of *Hoplobatrachus rugulosus*. The environmental variables used include: bio1 = annual mean temperature; bio4 = temperature seasonality; bio5 = maximum temperature of warmest month; bio6 = minimum temperature of coldest month; bio12 = annual precipitation; bio15 = precipitation seasonality; bio16 = precipitation of wettest quarter; bio17 = precipitation of driest quarter.

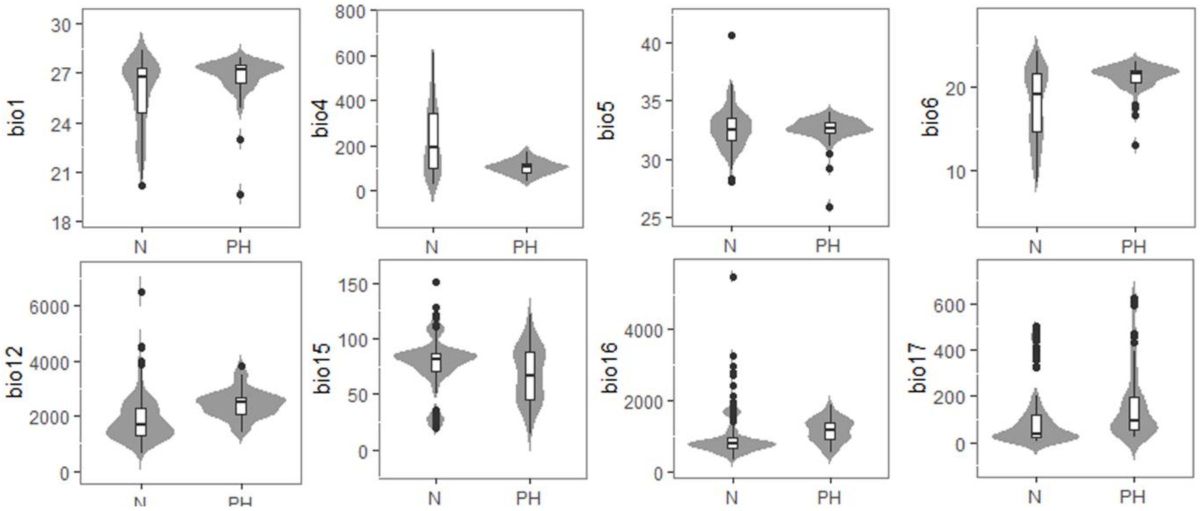

Fig. S4. Environmental variable scores (bar-and-whisker plots) and densities (violin plots) as observed at native (N) and Philippine-invaded range (PH) records of *Kaloula pulchra*. The environmental variables used include: bio1 = annual mean temperature; bio4 = temperature seasonality; bio5 = maximum temperature of warmest month; bio6 = minimum temperature of coldest month; bio12 = annual precipitation; bio15 = precipitation seasonality; bio16 = precipitation of wettest quarter; bio17 = precipitation of driest quarter.

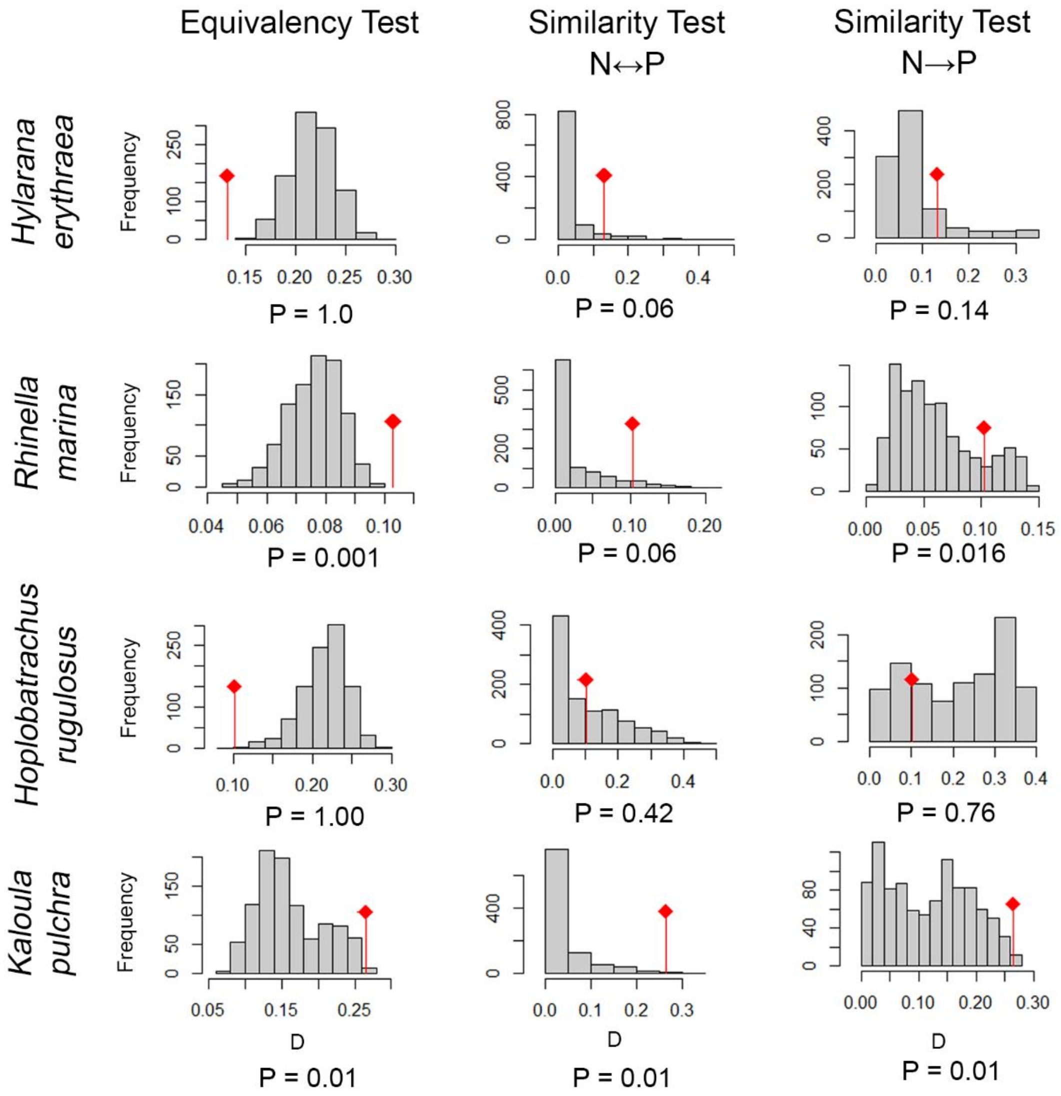

Fig. S5. Histograms showing the position of the observed Schoener's index of niche overlap *D* (red diamond flag) between species' (rows) native and Philippine niches within the null distribution of *D* values between two simulated random niches to test for niche equivalency and similarity (columns). Niche similarity test was conducted using two randomization methods: *N↔P* and *N→P* (see Methods). *P* values represent the quartile position of the observed *D* values within the null distribution.

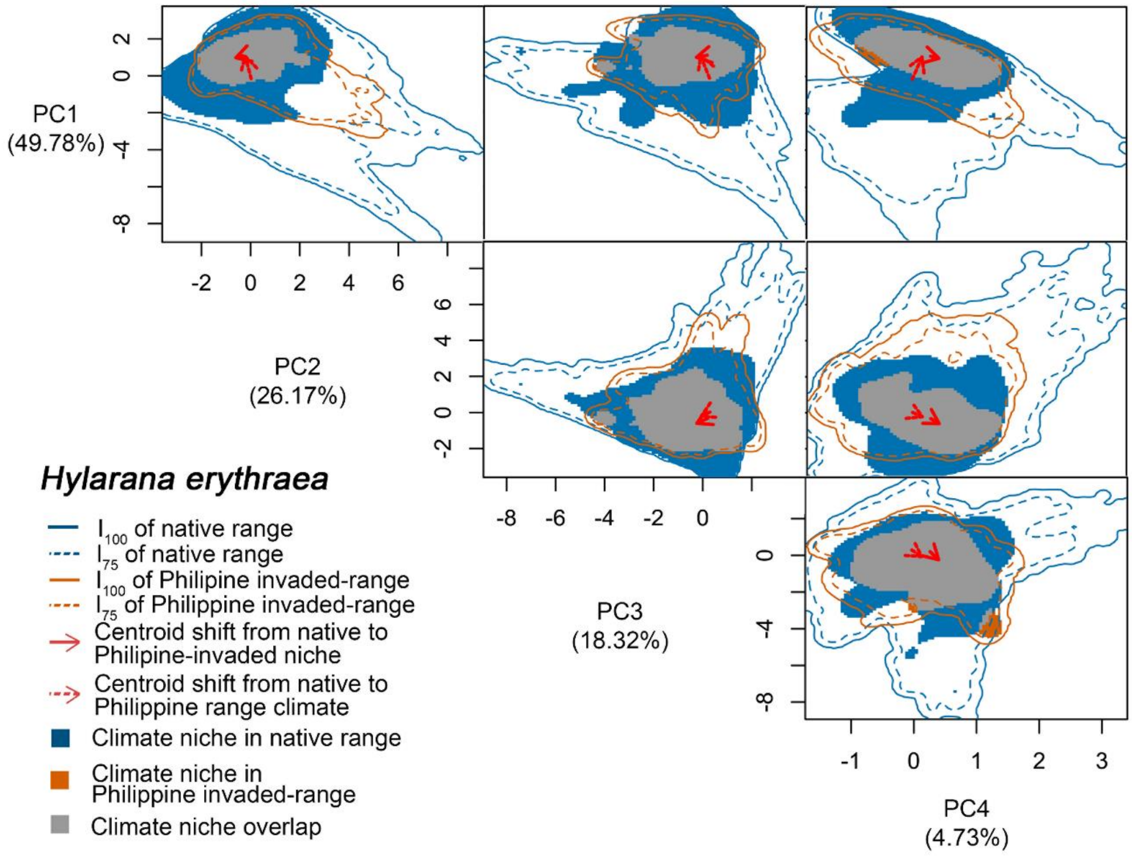

Fig. S6. The native (blue) and Philippine (red) niches of *Hylarana erythraea* as depicted in pairwise bi-plots of four Principal Component axes.

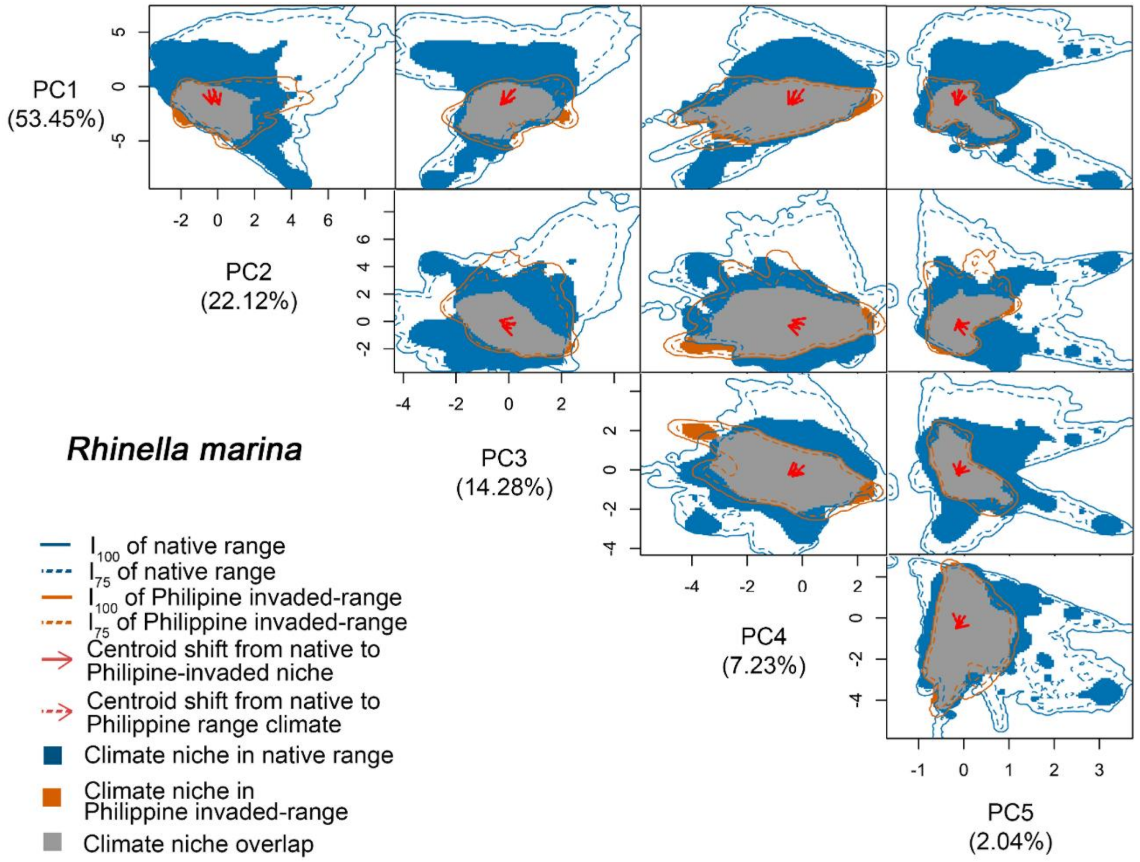

Fig. S7. The native (blue) and Philippine (red) niches of *Rhinella marina* as depicted in pairwise bi-plots of four Principal Component axes.

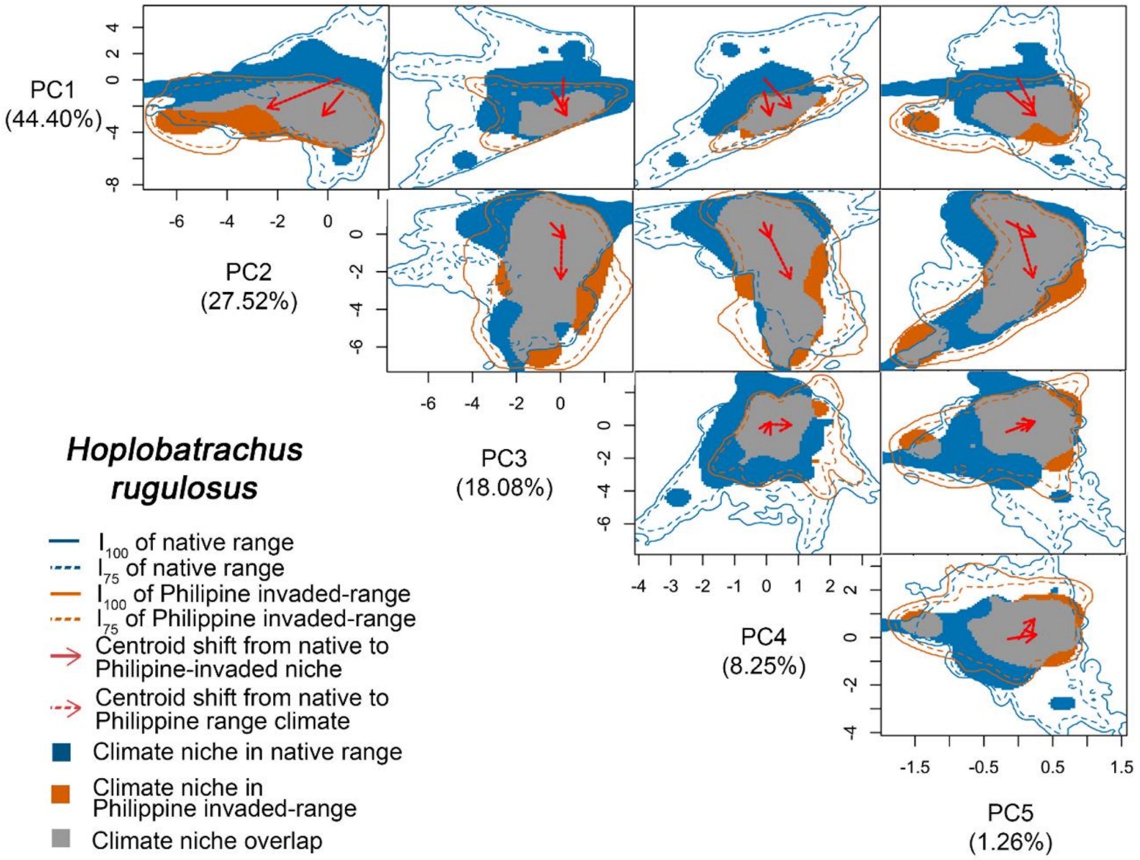

Fig. S8. The native (blue) and Philippine (red) niches of *Hoplobatrachus rugulosus* as depicted in pairwise bi-plots of four Principal Component axes.

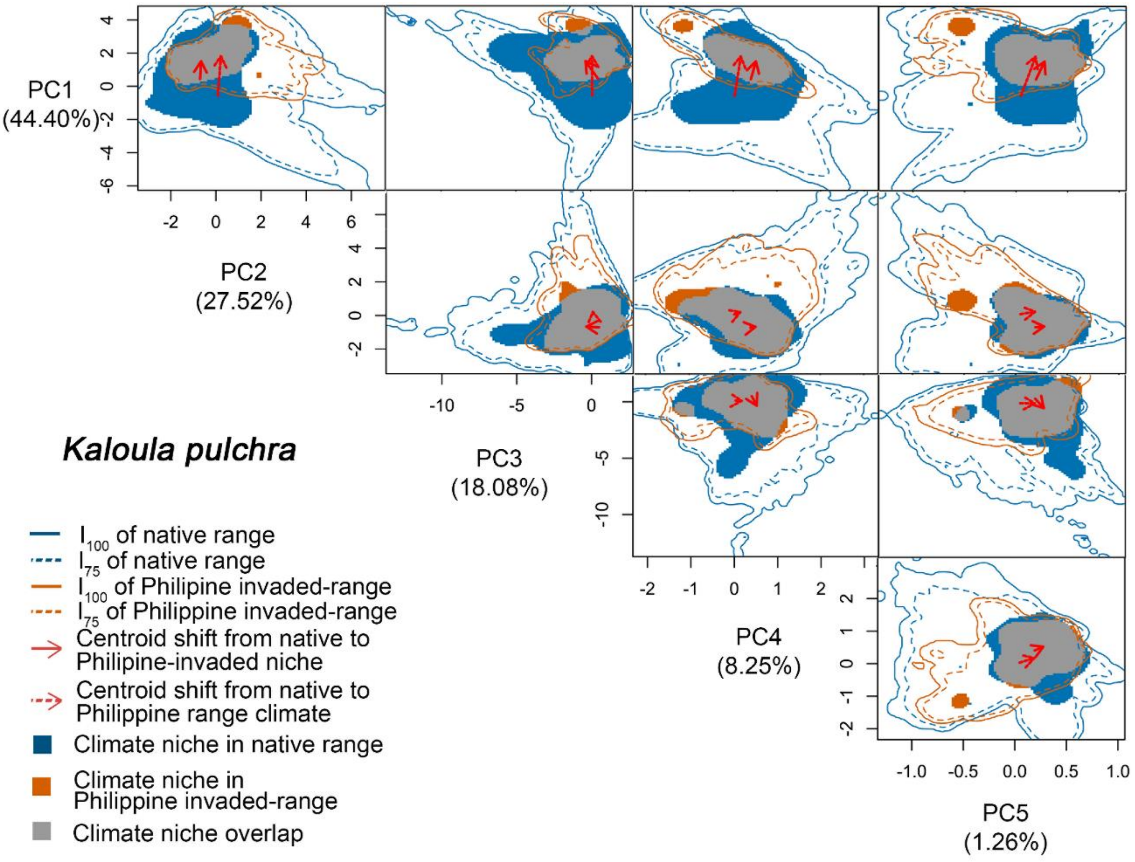

Fig. S9. The native (blue) and Philippine (red) niches of *Kaloula pulchra* as depicted in pairwise bi-plots of four Principal Component axes.

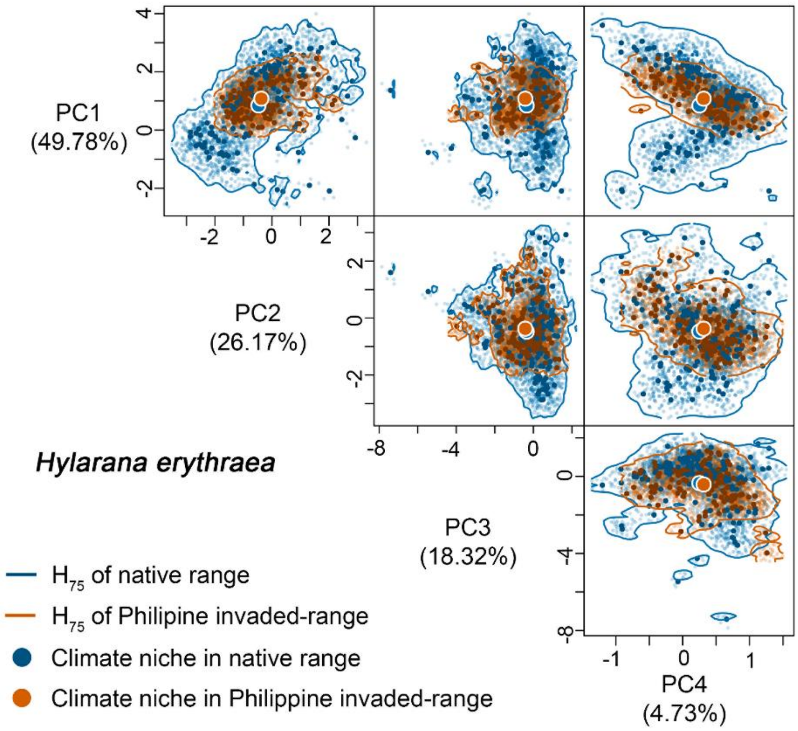

Fig. S10. The hypervolumes of the native niche (blue) and Philippine niche (red) of *Hylarana erythraea* in multidimensional environmental space defined by four PCs. The solid contour lines represent the 75% probability boundary of hypervolumes(H_{75}). The filled circles represent the centroids of the hypervolumes of the native niche (blue) and Philippine niche (red). Opaque dots represent true species' occurrence records, whereas transparent dots represent random records derived from Gaussian kernel density estimation.

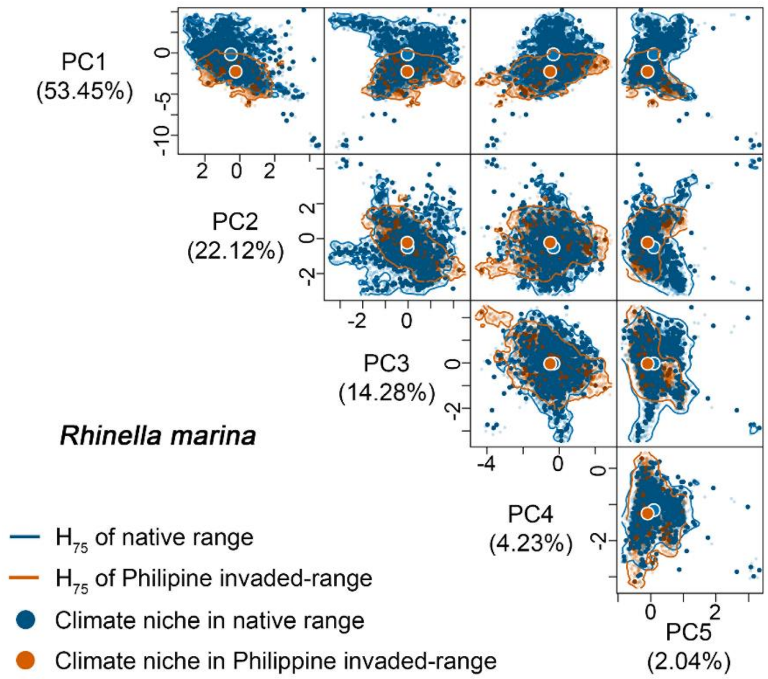

Fig. S11. The hypervolumes of the native niche (blue) and Philippine niche (red) of *Rhinella marina* in multidimensional environmental space defined by five PC. The solid contour lines represent the 75% probability boundary of hypervolumes(H_{75}). The filled circles represent the centroids of the hypervolumes of the native niche (blue) and Philippine niche (red). Opaque dots represent true species' occurrence records, whereas transparent dots represent random records derived from Gaussian kernel density estimation.

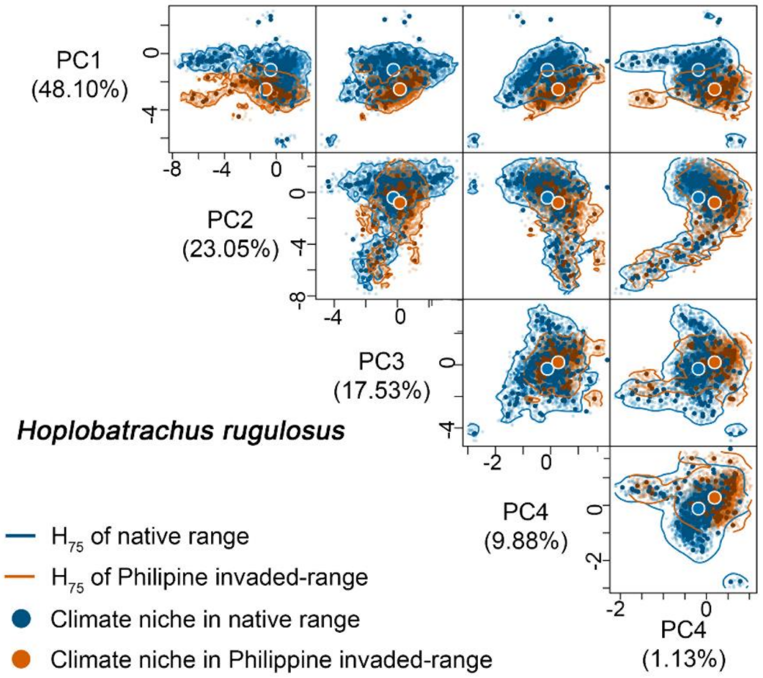

Fig. S12. The hypervolumes of the native niche (blue) and Philippine niche (red) of *Hoplobatrachus rugulosus* in multidimensional environmental space defined by five PC. The solid contour lines represent the 75% probability boundary of hypervolumes(H_{75}). The filled circles represent the centroids of the hypervolumes of the native niche (blue) and Philippine niche (red). Opaque dots represent true species' occurrence records, whereas transparent dots represent random records derived from Gaussian kernel density estimation.

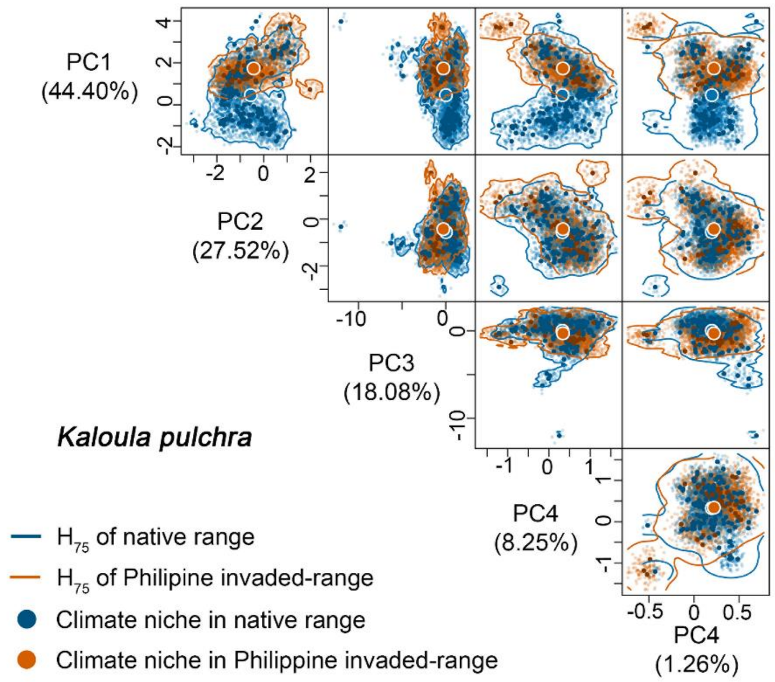

Fig. S13. The hypervolumes of the native niche (blue) and Philippine niche (red) of *Kaloula pulchra* in multidimensional environmental space defined by five PC. The solid contour lines represent the 75% probability boundary of hypervolumes(H_{75}). The filled circles represent the centroids of the hypervolumes of the native niche (blue) and Philippine niche (red). Bl Opaque dots represent true species' occurrence records, whereas transparent dots represent random records derived from Gaussian kernel density estimation.

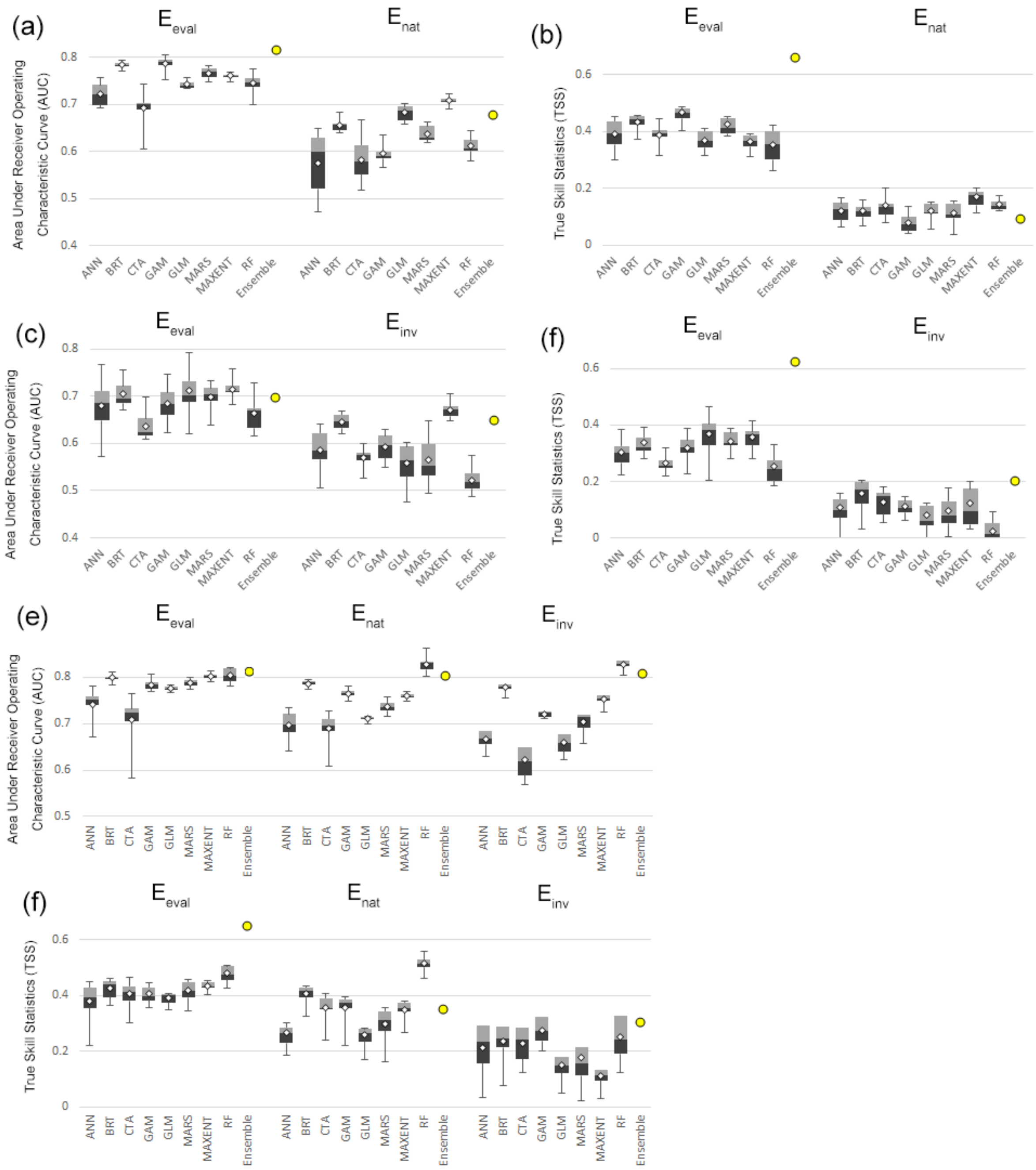

Fig. S14. Evaluation of the performance of the Philippine ecological niche models (ENMs) and ensemble models (EMs) (a, b), native ENMs and EMs (c, d), and combined range ENMs and EMs (e, f) of *Hylarana erythraea*, based on the AUC (a, c, e) and TSS (b, d, f). The box-and-whisker plots depict the minimum, first quartile, median, mean (open diamond), third quartile, and maximum values of the 10 replicates of each statistical technique. The scores of EMs are depicted as yellow circles. The statistical techniques used for modelling the ecological niche, as depicted on the x-axis include: Generalized Additive Model (GAM), Generalized Linear Model (GLM), Multivariate Adaptive Regression Splines (MARS), Classification and Regression Trees (CART), Artificial Neural Networks (ANN), Random Forests (RF), Boosted Regression Trees (BRT), and Maximum Entropy Model (Maxent). Ensembles of the ENMs were built using a weighted mean of probabilities algorithm.

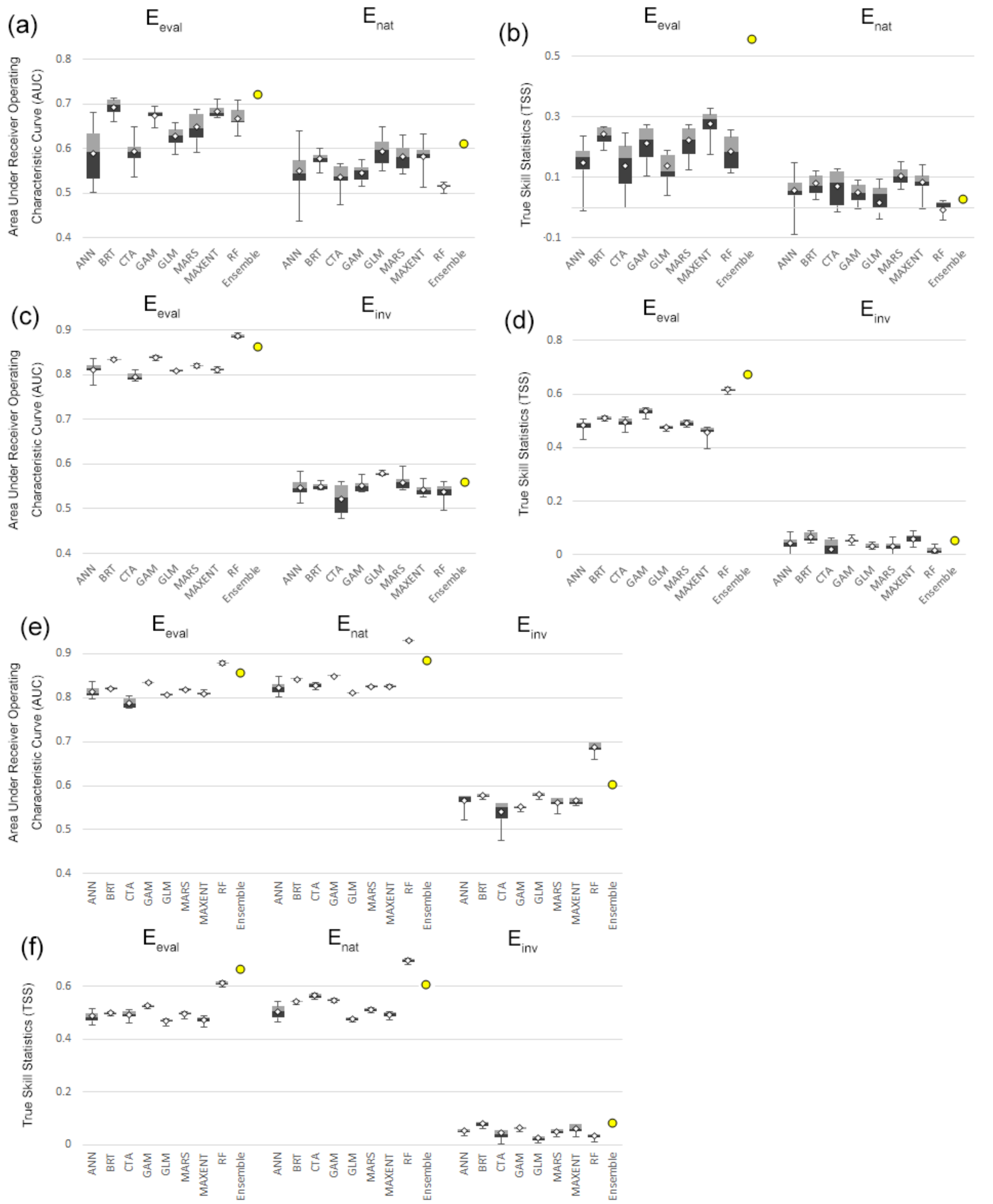

Fig. S15. Evaluation of the performance of the Philippine ecological niche models (ENMs) and ensemble models (EMs) (a, b), native ENMs and EMs (c, d), and combined range ENMs and EMs (e, f) of *Rhinella marina*, based on the AUC (a, c, e) and TSS (b, d, f). The box-and-whisker plots depict the minimum, first quartile, median, mean (open diamond), third quartile, and maximum values of the 10 replicates of each statistical technique. The scores of EMs are depicted as yellow circles. The statistical techniques used for modelling the ecological niche, as depicted on the x-axis include: Generalized Additive Model (GAM), Generalized Linear Model (GLM), Multivariate Adaptive Regression Splines (MARS), Classification and Regression Trees (CART), Artificial Neural Networks (ANN), Random Forests (RF), Boosted Regression Trees (BRT), and Maximum Entropy Model (Maxent). Ensembles of the ENMs were built using a weighted mean of probabilities algorithm.

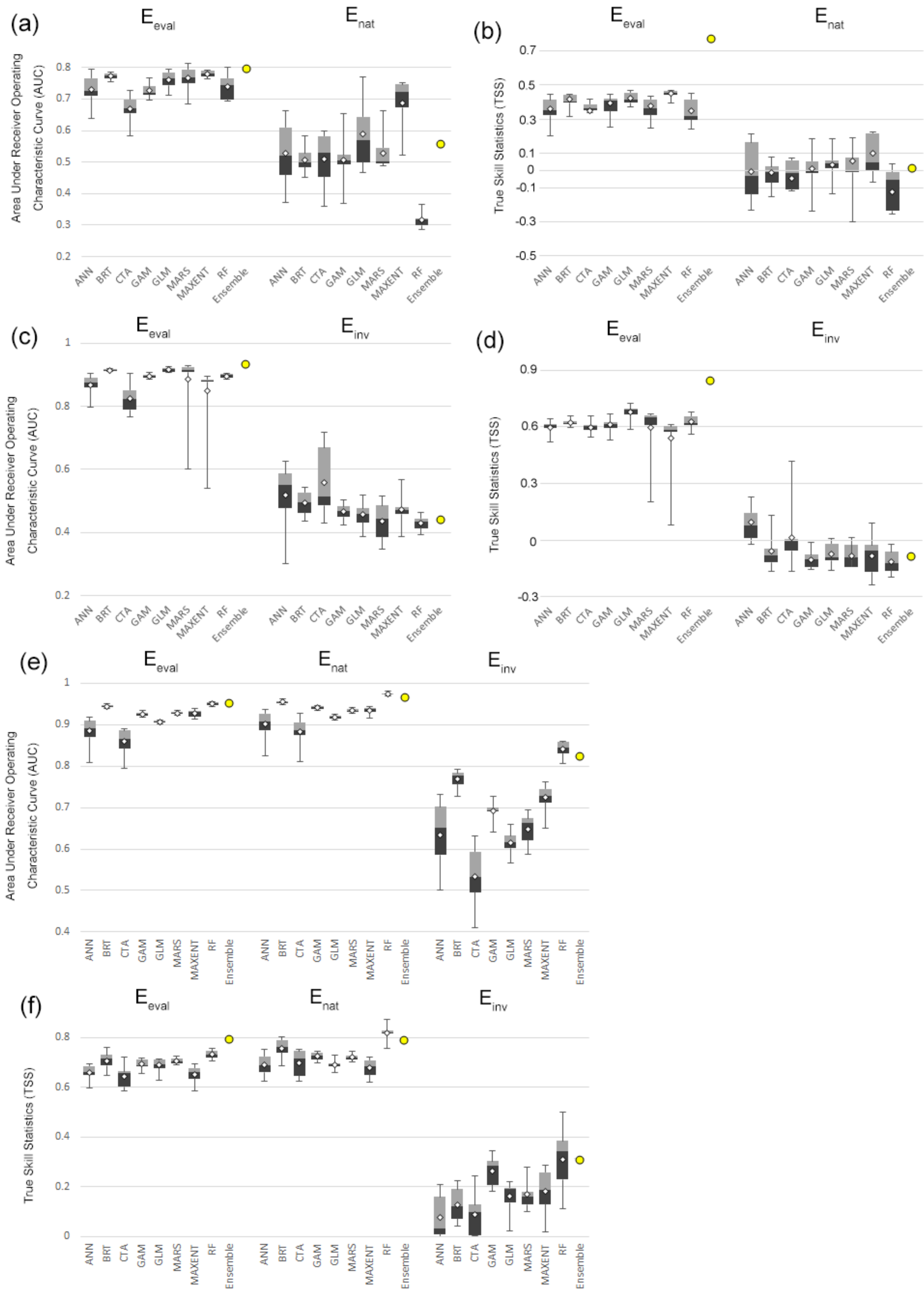

Fig. S16. Figure legend is on the next page.

Fig. S16. Evaluation of the performance of the Philippine ecological niche models (ENMs) and ensemble models (EMs) (a, b), native ENMs and EMs (c, d), and combined range ENMs and EMs (e, f) of *Hoplobatrachus rugulosus*, based on the AUC (a, c, e) and TSS (b, d, f). The box-and-whisker plots depict the minimum, first quartile, median, mean (open diamond), third quartile, and maximum values of the 10 replicates of each statistical technique. The scores of EMs are depicted as yellow circles. The statistical techniques used for modelling the ecological niche, as depicted on the x-axis include: Generalized Additive Model (GAM), Generalized Linear Model (GLM), Multivariate Adaptive Regression Splines (MARS), Classification and Regression Trees (CART), Artificial Neural Networks (ANN), Random Forests (RF), Boosted Regression Trees (BRT), and Maximum Entropy Model (Maxent). Ensembles of the ENMs were built using a weighted mean of probabilities algorithm.

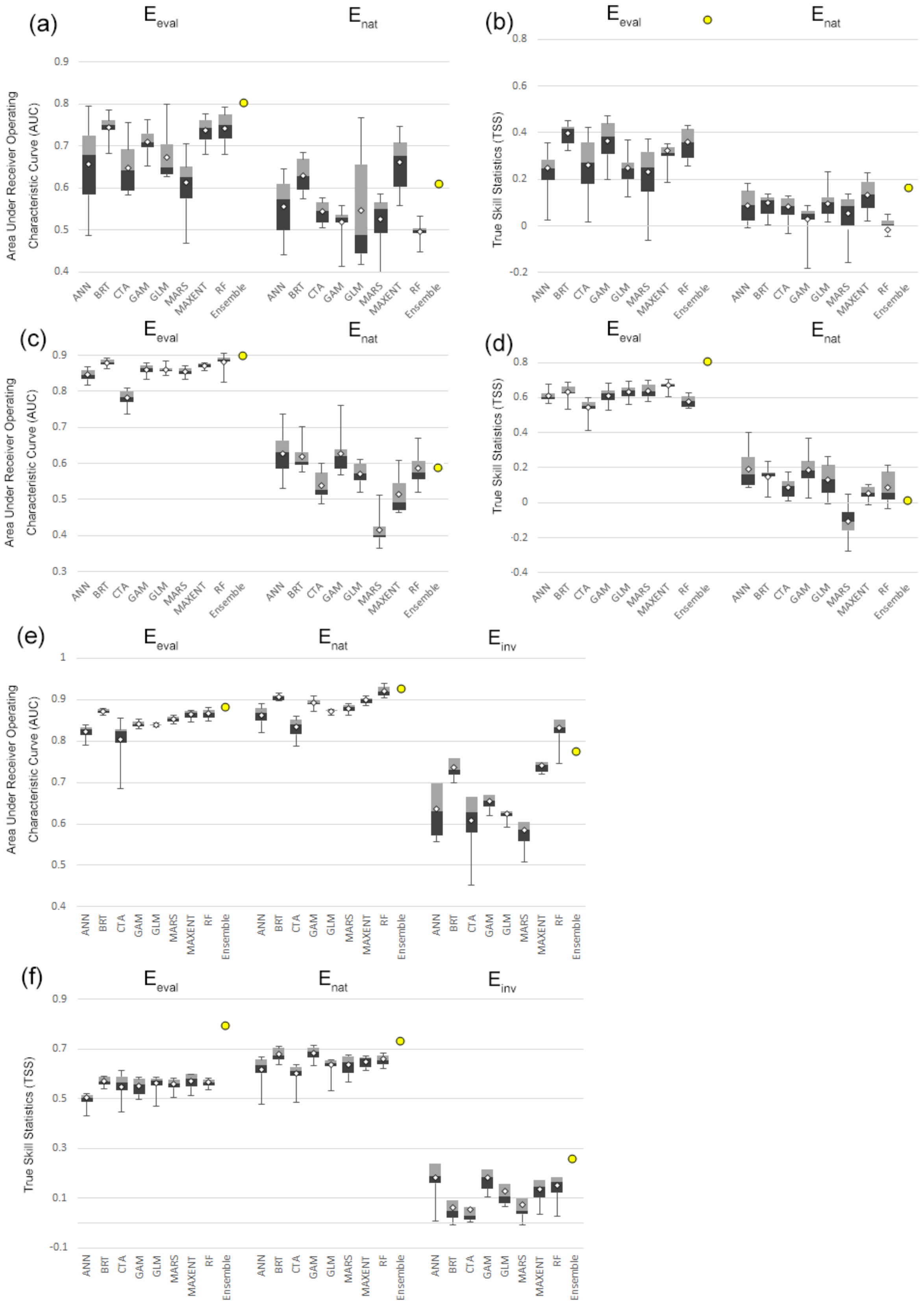

Fig. S17. Figure legend is on the next page.

Fig. S17. Evaluation of the performance of the Philippine ecological niche models (ENMs) and ensemble models (EMs) (a, b), native ENMs and EMs (c, d), and combined range ENMs and EMs (e, f) of *Kaloula pulchra*, based on the AUC (a, c, e) and TSS (b, d, f). The box-and-whisker plots depict the minimum, first quartile, median, mean (open diamond), third quartile, and maximum values of the 10 replicates of each statistical technique. The scores of EMs are depicted as yellow circles. The statistical techniques used for modelling the ecological niche, as depicted on the x-axis include: Generalized Additive Model (GAM), Generalized Linear Model (GLM), Multivariate Adaptive Regression Splines (MARS), Classification and Regression Trees (CART), Artificial Neural Networks (ANN), Random Forests (RF), Boosted Regression Trees (BRT), and Maximum Entropy Model (Maxent). Ensembles of the ENMs were built using a weighted mean of probabilities algorithm.

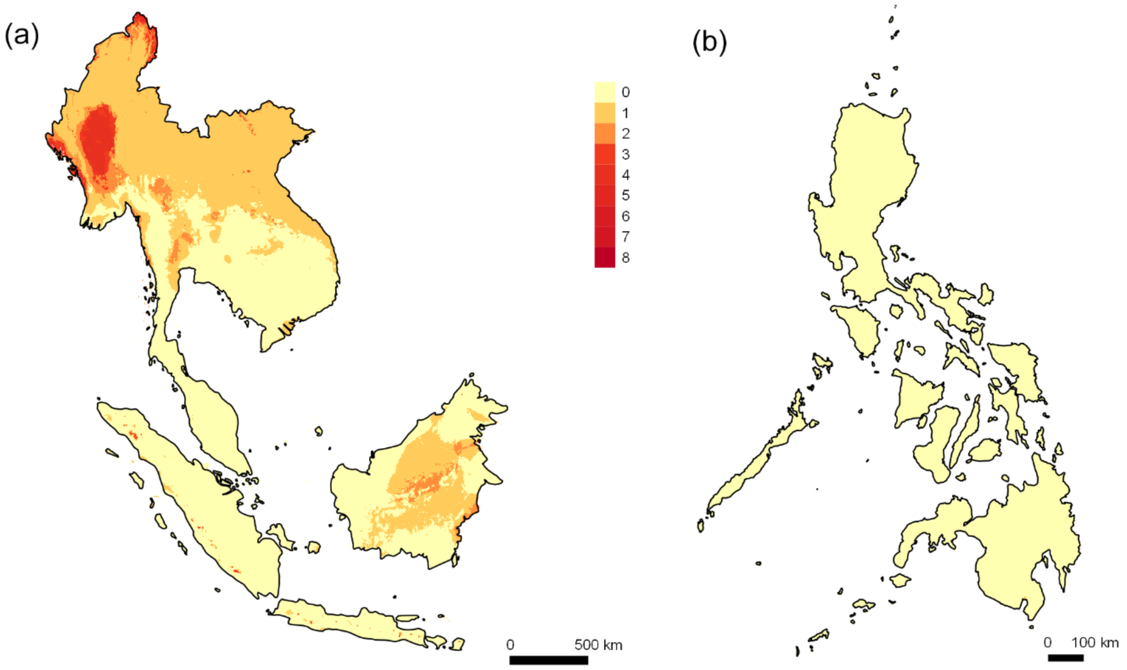

Fig. S18. Clamping of environmental variables when the variable values used for calibrating Philippine ENMs of *Hylarana erythraea* are projected to its native range (Philippine ENMs → native ENMs) (a) and vice-versa (b). Hotter colors (red) indicate locations where at least one environmental variable has values outside of the range of that used for calibrating models. High levels of clamping of environmental variables translate to higher uncertainty of predictions. The maps were created using QGIS Geographic Information System software (v. 3.14; http://qgis.osgeo.org) and projected using WGS 1984 Coordinate Reference System.

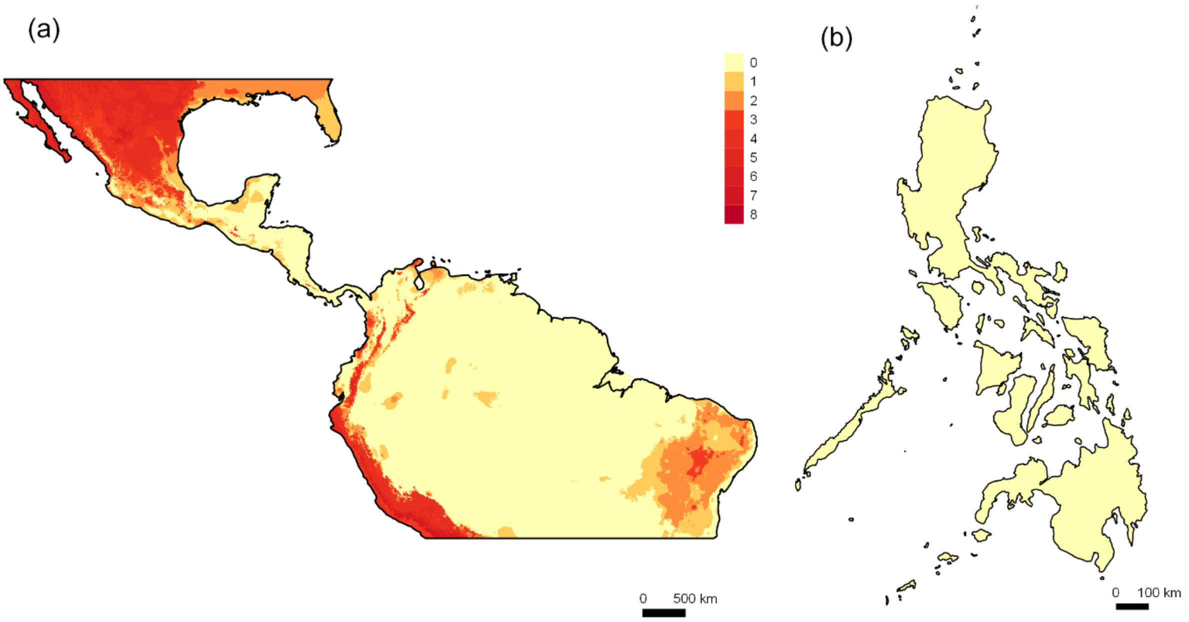

Fig. S19. Clamping of environmental variables when the variable values used for calibrating Philippine ENMs of *Rhinella marina* are projected to its native range (Philippine ENMs → native ENMs) (a) and vice-versa (b). Hotter colors (red) indicate locations where at least one environmental variable has values outside of the range of that used for calibrating models. High levels of clamping of environmental variables translate to higher uncertainty of predictions. The maps were created using QGIS Geographic Information System software (v. 3.14; http://qgis.osgeo.org) and projected using WGS 1984 Coordinate Reference System.

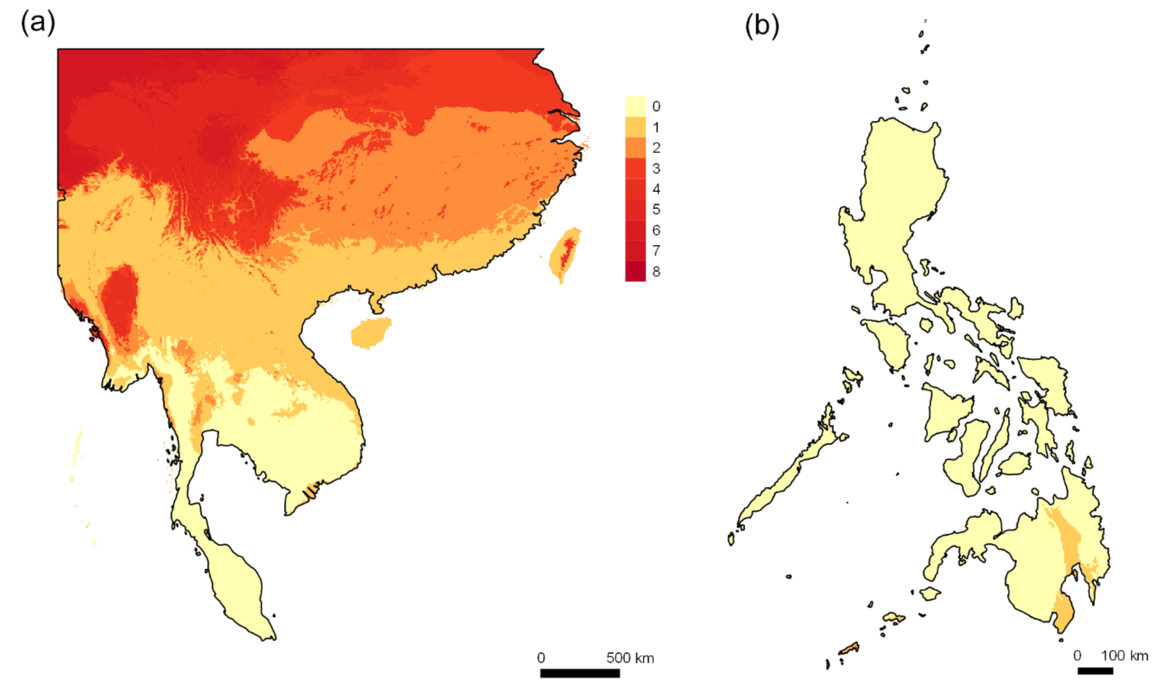

Fig. S20. Clamping of environmental variables when the variable values used for calibrating Philippine ENMs of *Hoplobatrachus rugulosus* are projected to its native range (Philippine ENMs → native ENMs) (a) and vice-versa (b). Hotter colors (red) indicate locations where at least one environmental variable has values outside of the range of that used for calibrating models. High levels of clamping of environmental variables translate to higher uncertainty of predictions. The maps were created using QGIS Geographic Information System software (v. 3.14; http://qgis.osgeo.org) and projected using WGS 1984 Coordinate Reference System.

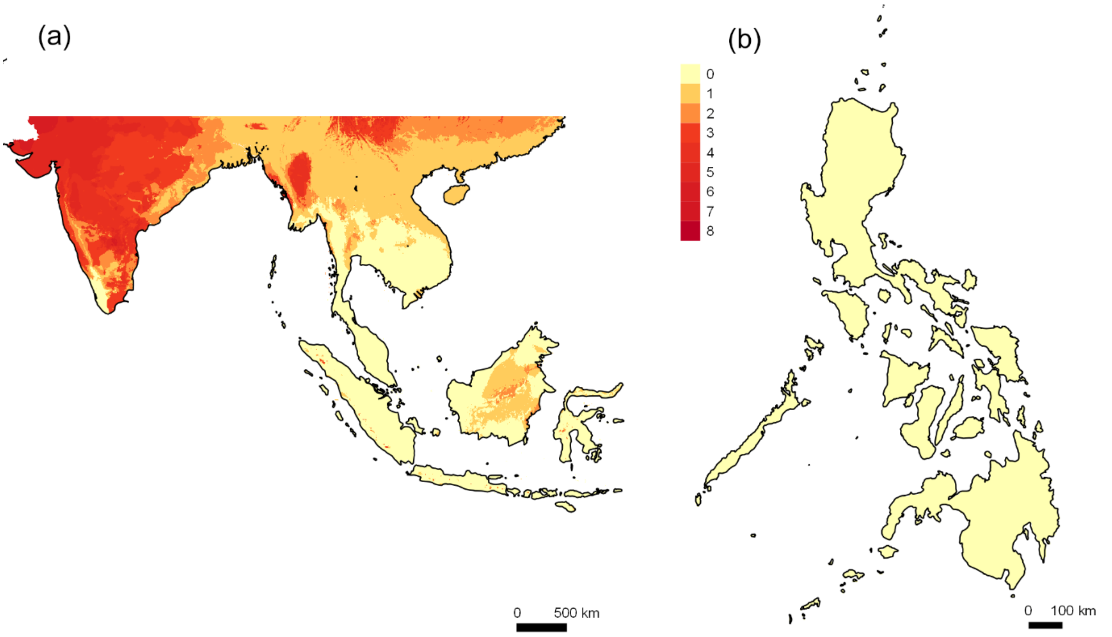

Fig. S21. Clamping of environmental variables when the variable values used for calibrating Philippine ENMs of *Kaloula pulchra* are projected to its native range (Philippine ENMs → native ENMs) (a) and vice-versa (b). Hotter colors (red) indicate locations where at least one environmental variable has values outside of the range of that used for calibrating models. High levels of clamping of environmental variables translate to higher uncertainty of predictions. The maps were created using QGIS Geographic Information System software (v. 3.14; http://qgis.osgeo.org) and projected using WGS 1984 Coordinate Reference System

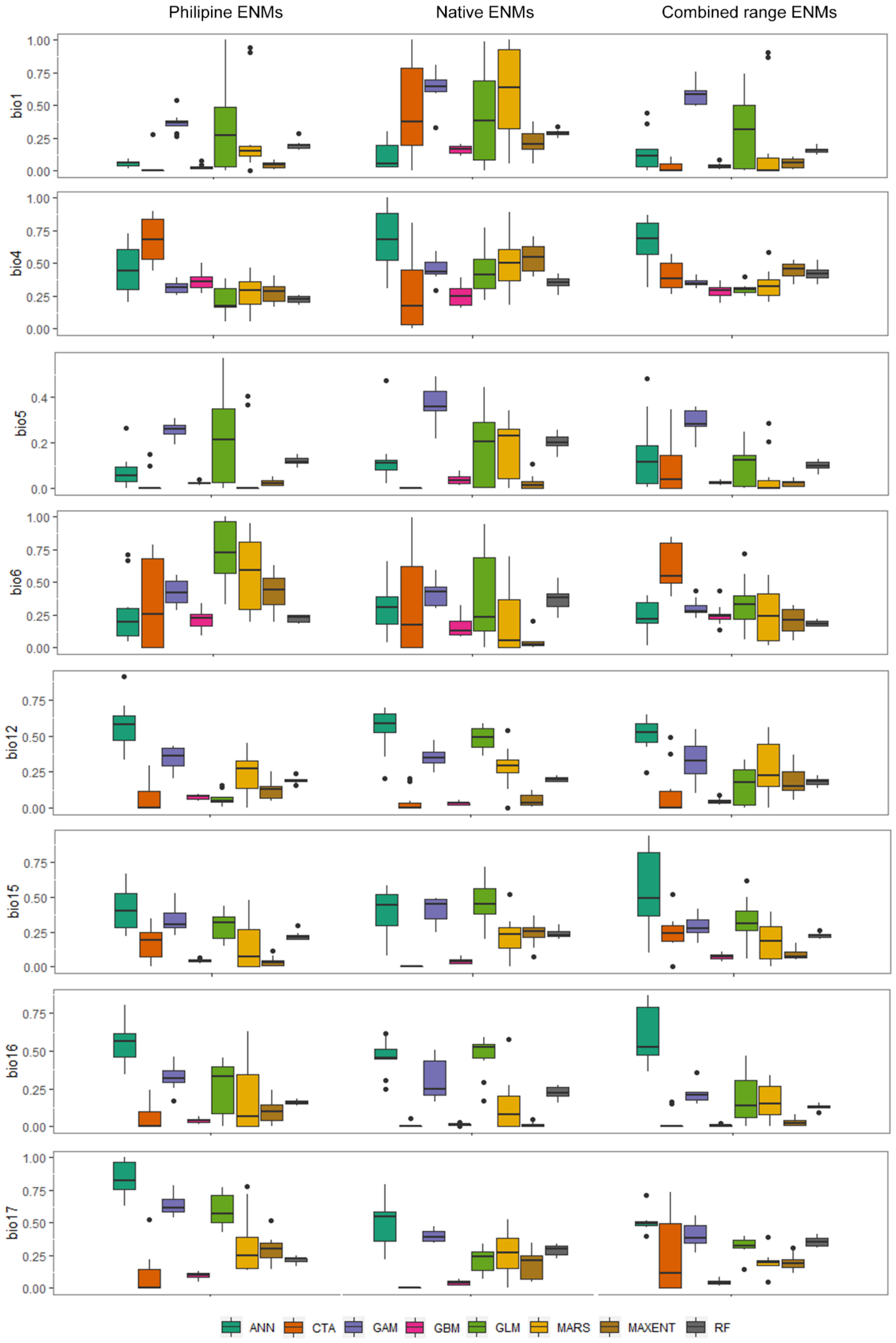

Fig. S22. Figure legend is on the next page.

Fig. S22. Relative variable contribution of eight environmental variables in eighty ecological niche models (ten replicates per eight statistical techniques) calibrated either using data from the Philippine-invaded range (Philippine ENMs), native range (native ENMs), and combined Philippine-invaded and native ranges (combined range ENMs) of *Hylarana erythraea*. The eight statistical techniques used include: Generalized Additive Model (GAM), Generalized Linear Model (GLM), Multivariate Adaptive Regression Splines (MARS), Classification and Regression Trees (CART), Artificial Neural Networks (ANN), Random Forests (RF), Boosted Regression Trees (BRT), and Maximum Entropy Model (Maxent). Ensembles of the ENMs were built using a weighted mean of probabilities algorithm. The environmental variables used include environmental variables: bio1 = annual mean temperature; bio4 = temperature seasonality; bio5 = maximum temperature of warmest month; bio6 = minimum temperature of coldest month; bio12 = annual precipitation; bio15 = precipitation seasonality; bio16 = precipitation of wettest quarter; bio17 = precipitation of driest quarter.

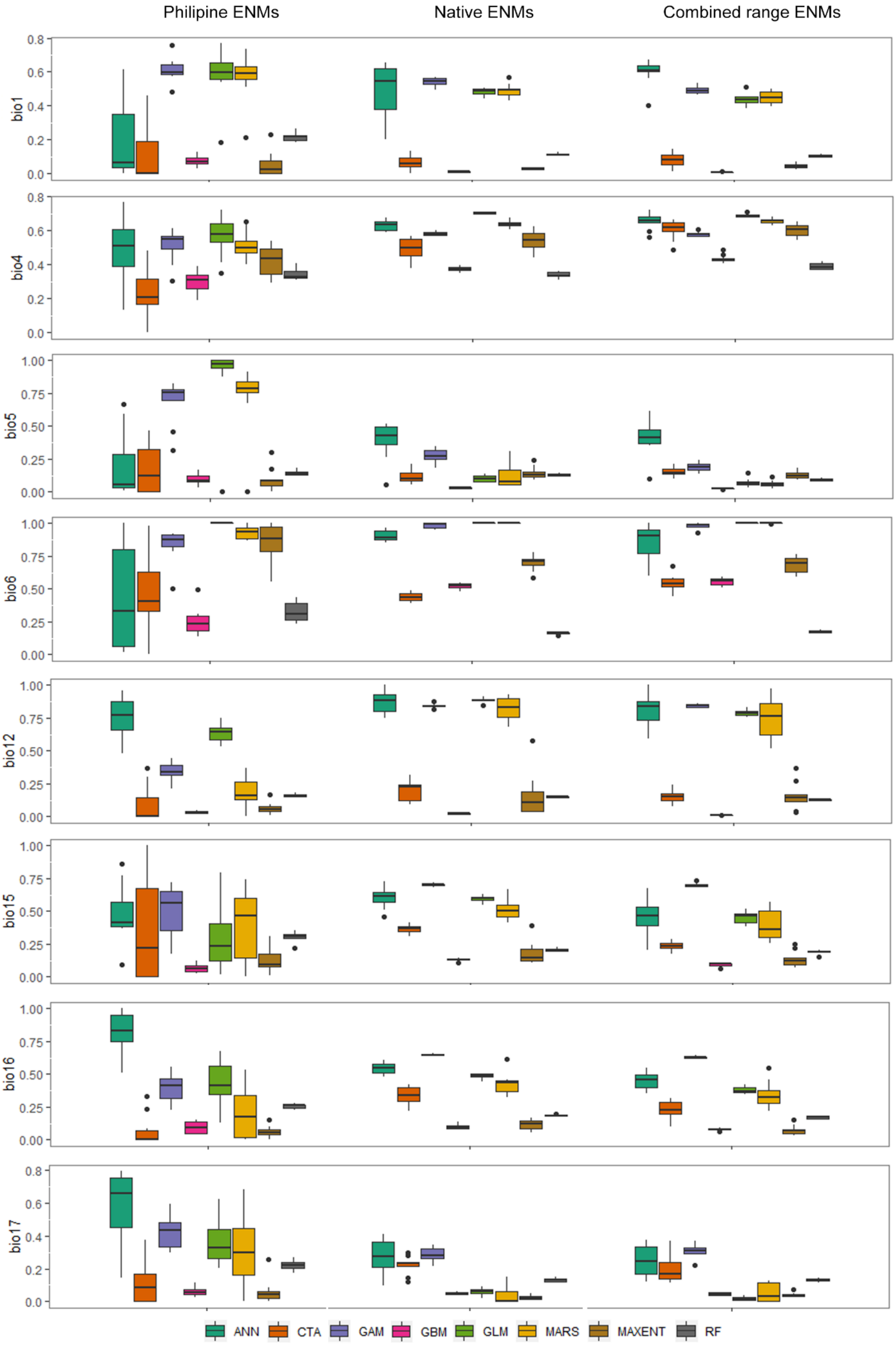

Fig. S23. Figure legend is on the next page.

Fig. S23. Relative variable contribution of eight environmental variables in eighty ecological niche models (ten replicates per eight statistical techniques) calibrated either using data from the Philippine-invaded range (Philippine ENMs), native range (native ENMs), and combined Philippine-invaded and native ranges (combined range ENMs) of *Rhinella marina*. The eight statistical techniques used include: Generalized Additive Model (GAM), Generalized Linear Model (GLM), Multivariate Adaptive Regression Splines (MARS), Classification and Regression Trees (CART), Artificial Neural Networks (ANN), Random Forests (RF), Boosted Regression Trees (BRT), and Maximum Entropy Model (Maxent). Ensembles of the ENMs were built using a weighted mean of probabilities algorithm. The environmental variables used include environmental variables: bio1 = annual mean temperature; bio4 = temperature seasonality; bio5 = maximum temperature of warmest month; bio6 = minimum temperature of coldest month; bio12 = annual precipitation; bio15 = precipitation seasonality; bio16 = precipitation of wettest quarter; bio17 = precipitation of driest quarter.

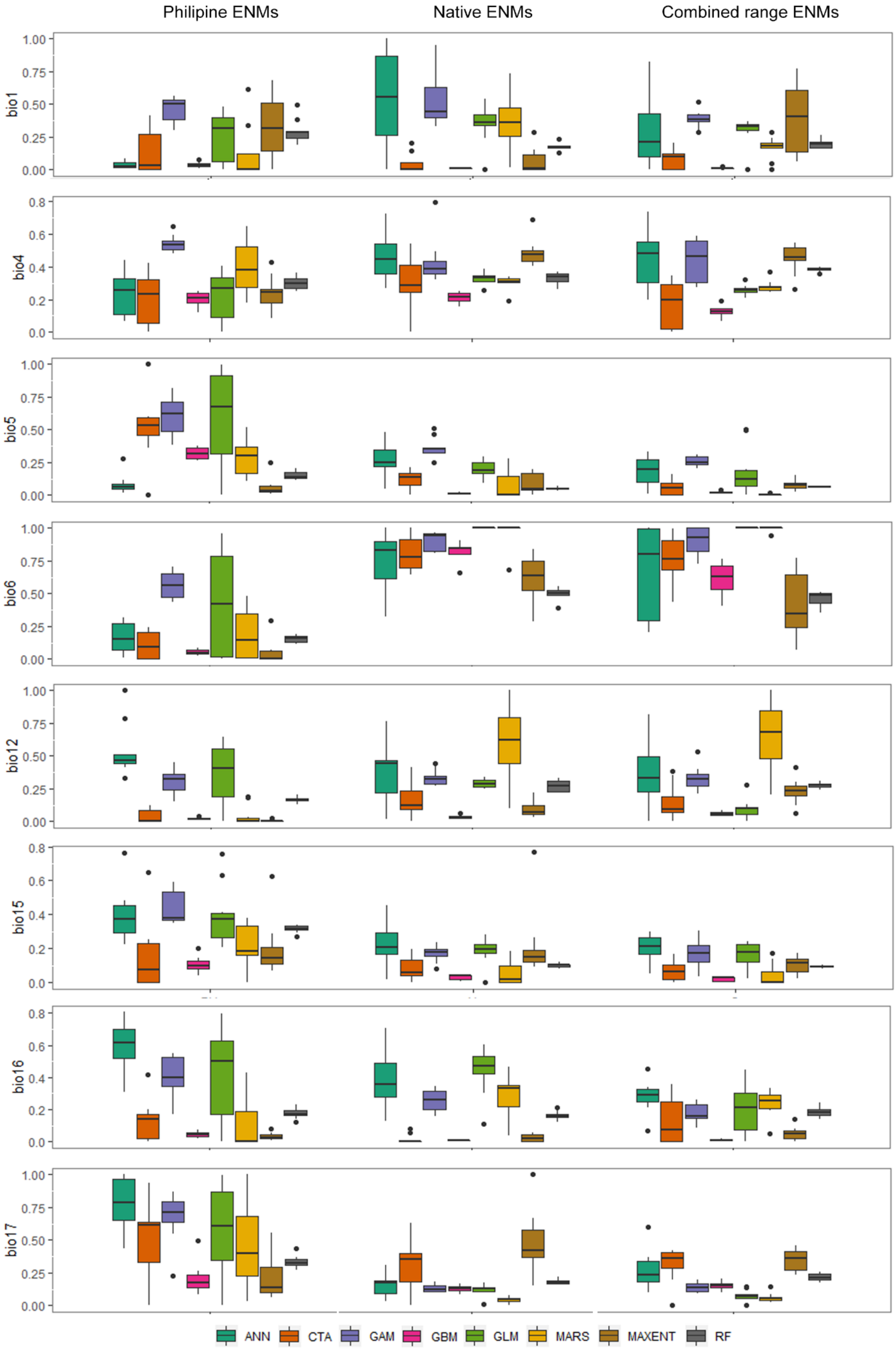

Fig. S24. Figure legend is on the next page.

Fig. S24. Relative variable contribution of eight environmental variables in eighty ecological niche models (ten replicates per eight statistical techniques) calibrated either using data from the Philippine-invaded range (Philippine ENMs), native range (native ENMs), and combined Philippine-invaded and native ranges (combined range ENMs) of *Hoplobatrachus rugulosus*. The eight statistical techniques used include: Generalized Additive Model (GAM), Generalized Linear Model (GLM), Multivariate Adaptive Regression Splines (MARS), Classification and Regression Trees (CART), Artificial Neural Networks (ANN), Random Forests (RF), Boosted Regression Trees (BRT), and Maximum Entropy Model (Maxent). Ensembles of the ENMs were built using a weighted mean of probabilities algorithm. The environmental variables used include environmental variables: bio1 = annual mean temperature; bio4 = temperature seasonality; bio5 = maximum temperature of warmest month; bio6 = minimum temperature of coldest month; bio12 = annual precipitation; bio15 = precipitation seasonality; bio16 = precipitation of wettest quarter; bio17 = precipitation of driest quarter.

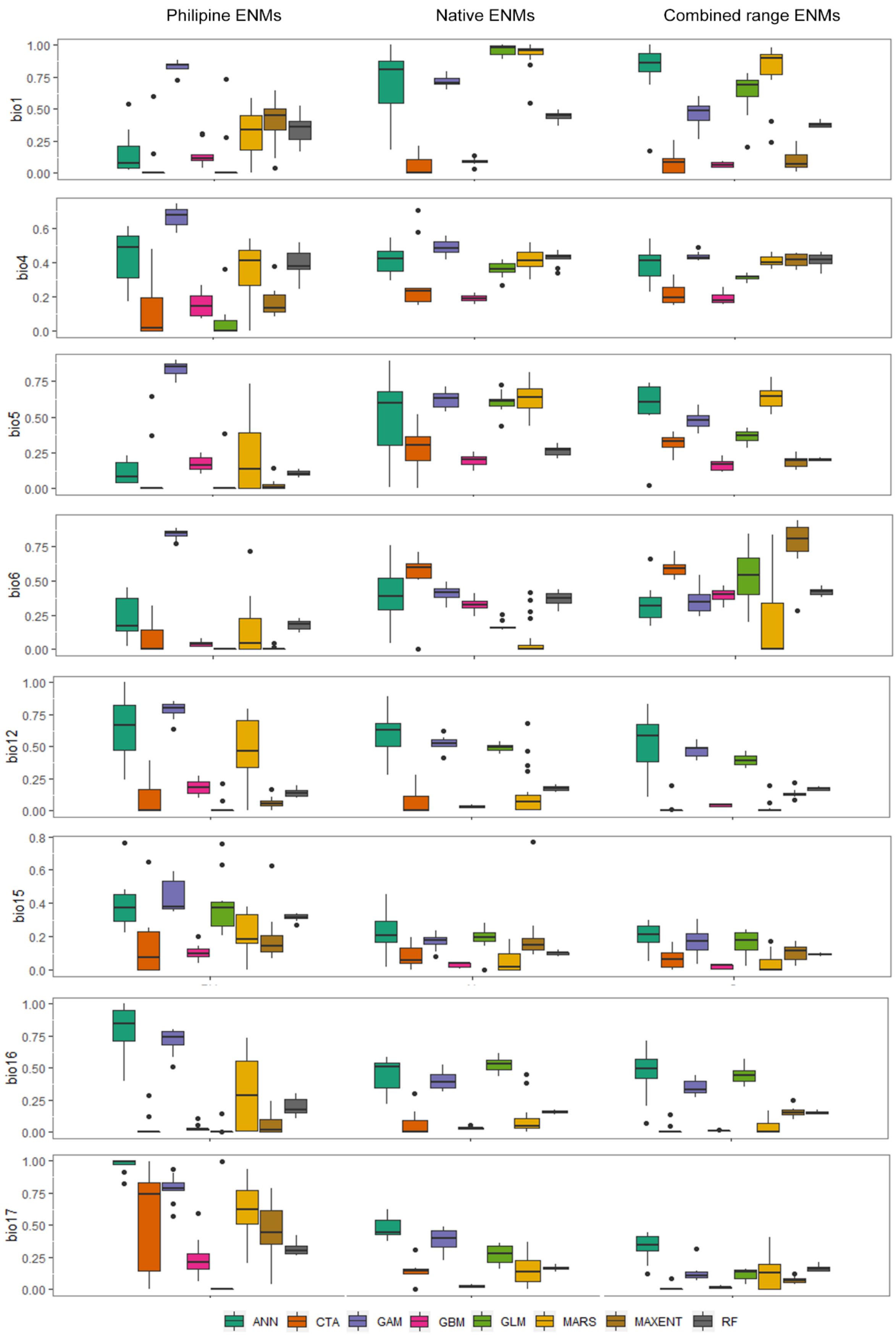

Fig.S25. Figure legend is on the next page.

Fig. S25. Relative variable contribution of eight environmental variables in eighty ecological niche models (ten replicates per eight statistical techniques) calibrated either using data from the Philippine-invaded range (Philippine ENMs), native range (native ENMs), and combined Philippine-invaded and native ranges (combined range ENMs) of *Kaloula pulchra*. The eight statistical techniques used include: Generalized Additive Model (GAM), Generalized Linear Model (GLM), Multivariate Adaptive Regression Splines (MARS), Classification and Regression Trees (CART), Artificial Neural Networks (ANN), Random Forests (RF), Boosted Regression Trees (BRT), and Maximum Entropy Model (Maxent). Ensembles of the ENMs were built using a weighted mean of probabilities algorithm. The environmental variables used include environmental variables: bio1 = annual mean temperature; bio4 = temperature seasonality; bio5 = maximum temperature of warmest month; bio6 = minimum temperature of coldest month; bio12 = annual precipitation; bio15 = precipitation seasonality; bio16 = precipitation of wettest quarter; bio17 = precipitation of driest quarter.

Table S1. Eigenvalues of Principal Component axes and correlation of eight environmental variables[1] to Principal Component axes. bio1 = annual mean temperature; bio4 = temperature seasonality; bio5 = maximum temperature of warmest month; bio6 = minimum temperature of coldest month; bio12 = annual precipitation; bio15 = precipitation seasonality; bio16 = precipitation of wettest quarter; bio17 = precipitation of driest quarter.

	PC1	PC2	PC3	PC4	PC5
Hylarana erythraea					
% Variance	49.78%	26.17%	18.32%	4.73%	-
Correlation					
bio1	0.72	-0.66	-0.18	-0.01	-
bio4	-0.92	-0.07	-0.49	-0.10	-
bio5	0.36	-0.84	-0.46	0.13	-
bio6	0.90	-0.37	-0.93	0.05	-
bio12	0.64	0.58	-0.93	-0.28	-
bio15	-0.80	-0.34	0.19	-0.35	-
bio16	0.05	0.35	-0.13	-0.32	-
bio17	0.80	0.48	-0.08	0.21	-
Rhinella marina					
% Variance	53.45%	22.12%	14.28%	7.23%	2.04%
Correlation					
bio1	-0.75	-0.63	-0.11	0.16	0.01
bio4	0.73	-0.02	-0.62	-0.25	-0.03
bio5	-0.15	-0.81	-0.55	-0.09	0.01
bio6	-0.88	-0.34	0.17	0.24	0.02
bio12	-0.92	0.24	-0.05	-0.29	-0.03
bio15	0.59	-0.53	0.48	-0.30	0.25
bio16	-0.84	-0.01	0.19	-0.50	-0.10
bio17	-0.70	0.52	-0.39	0.02	0.30

Table S1. continued

	PC1	PC2	PC3	PC4	PC5
Hoplobatrachus rugulosus					
% Variance	48.10%	23.05%	17.53%	9.88%	1.13%
Correlation					
bio1	-0.92	-0.03	0.40	0.02	-0.01
bio4	0.87	-0.06	-0.07	-0.48	-0.01
bio5	-0.42	-0.18	0.65	-0.60	-0.01
bio6	-0.94	-0.04	0.25	0.22	0.02
bio12	-0.71	-0.37	-0.56	-0.18	0.08
bio15	-0.41	0.86	-0.20	-0.10	-0.20
bio16	-0.72	0.02	-0.62	-0.31	0.02
bio17	-0.02	-0.97	-0.11	0.10	-0.21
Kaloula pulchra					
% Variance	44.40%	27.52%	18.08%	8.25%	1.26%
Correlation					
bio1	0.68	-0.71	0.16	-0.04	0.05
bio4	-0.88	0.09	-0.14	-0.43	0.10
bio5	0.05	-0.85	0.15	-0.50	-0.01
bio6	0.88	-0.39	0.16	0.19	0.06
bio12	0.76	0.30	-0.54	-0.17	0.03
bio15	-0.54	-0.67	-0.42	0.17	-0.20
bio16	0.36	-0.10	-0.92	-0.04	0.04
bio17	0.72	0.52	0.19	-0.35	-0.20

[1]bio1 = annual mean temperature; bio4 = temperature seasonality; bio5 = maximum temperature of warmest month; bio6 = minimum temperature of coldest month; bio12 = annual precipitation; bio15 = precipitation seasonality; bio16 = precipitation of wettest quarter; bio17 = precipitation of driest quarter.

Table S2. Results of overlap index, niche equivalency and similarity tests, and niche change metrics of realized climatic niches of the four alien amphibian species in their Philippine-invaded ranges.

Principal Component Axes	Total Variance %	Overlap Schoener's *D*	Conservatism						Niche change metrics					
			Niche Equivalency		Niche Similarity				Stability		Unfilling		Expansion	
					N↔P		N→P							
			P value	cohen's *d*	*P* value	cohen's *d*	*P* value	cohen's *d*	I_{75} %	I_{100} %	I_{75} %	I_{100} %	I_{75} %	I_{100} %
Hylarana erythraea														
PC1 x PC2	75.95	0.13	1.00	3.91	0.06	1.84	0.14	0.68	100	100	24	30	0	0
PC1 x PC3	68.11	0.20	0.99	2.34	0.09	1.72	0.29	0.55	100	100	33	38	0	0
PC1 x PC4	54.51	0.07	1.00	3.22	0.23	0.58	0.20	0.84	99	99	20	27	1	1
PC2 x PC3	44.50	0.06	1.00	3.24	0.27	0.05	0.29	0.00	100	100	14	19	0	0
PC2 x PC4	30.90	0.11	1.00	6.21	0.20	0.64	0.07	1.38	100	100	21	23	0	0
PC3 x PC4	23.06	0.33	0.53	0.08	0.02	2.35	0.09	1.50	100	99	10	13	0	1
Rhinella marina														
PC1 x PC2	75.58	0.10	0.01	2.97	0.07	1.90	0.12	1.30	100	100	7	13	0	0
PC1 x PC3	67.74	0.19	0.001	3.32	0.10	1.56	0.55	0.00	100	100	7	17	0	0
PC1 x PC4	60.68	0.21	0.07	1.58	0.08	1.65	0.38	0.39	100	99	1	3	0	1
PC1 x PC5	55.49	0.04	1.00	2.97	0.12	1.12	0.40	0.19	100	100	5	12	0	0
PC2 x PC3	36.41	0.05	1.00	4.51	0.25	0.53	0.07	1.20	100	100	12	14	0	0
PC3 x PC4	29.35	0.13	0.18	0.89	0.31	0.39	0.54	0.12	100	100	2	3	0	0
PC2 x PC5	24.16	0.08	0.001	4.25	0.06	2.28	0.12	1.79	100	100	4	7	0	0
PC3 x PC4	21.51	0.20	0.09	1.42	0.07	1.77	0.37	0.51	100	99	1	2	0	1
PC3 x PC5	16.32	0.09	1.00	4.34	0.01	2.97	0.01	1.60	100	100	3	6	0	0
PC4 x PC5	9.26	0.18	0.001	3.15	0.13	1.43	0.42	0.35	100	100	1	2	0	0

Table S2. continued

Principal Component Axes	Total Variance %	Overlap Schoenner's *D*	Conservatism						Niche change metrics					
			Niche Equivalency		Niche Similarity				Stability		Unfilling		Expansion	
					N↔P		N→P							
			P value	cohen's *d*	*P* value	cohen's *d*	*P* value	cohen's d	I_{75} %	I_{100} %	I_{75} %	I_{100} %	I_{75} %	I_{100} %
Hoplobatrachus rugulosus														
PC1 x PC2	71.15	0.10	1.00	3.97	0.42	0.01	0.75	0.97	93	91	6	16	7	9
PC1 x PC3	65.63	0.16	0.86	1.08	0.03	2.63	0.001	2.89	100	100	55	62	0	0
PC1 x PC4	57.98	0.14	0.001	3.39	0.10	1.51	0.02	1.93	97	97	8	28	3	3
PC1 x PC5	49.23	0.04	1.00	5.06	0.37	0.10	0.52	0.35	90	88	29	39	10	12
PC2 x PC3	40.58	0.14	0.77	0.72	0.39	0.23	0.47	0.03	98	97	7	11	2	3
PC2 x PC4	32.93	0.12	0.31	0.53	0.31	0.45	0.26	0.79	97	97	3	6	3	3
PC2 x PC5	24.18	0.14	0.36	0.40	0.09	1.27	0.08	1.49	100	99	4	5	0	1
PC3 x PC4	27.41	0.09	1.00	4.59	0.12	1.32	0.24	0.53	99	99	19	22	1	1
PC3 x PC5	18.66	0.08	1.00	2.85	0.49	0.17	0.37	0.15	97	96	25	27	3	4
PC4 x PC5	11.01	0.10	0.87	1.12	0.21	0.69	0.20	0.81	98	97	19	23	2	3
Kaloula pulchra														
PC1 x PC2	71.92	0.26	0.01	2.27	0.01	4.12	0.01	2.01	98	98	10	18	2	2
PC1 x PC3	62.49	0.08	0.99	2.07	0.18	0.90	0.21	0.82	97	97	16	27	3	3
PC1 x PC4	52.65	0.18	0.33	0.16	0.05	2.21	0.01	1.57	98	98	9	17	2	2
PC1 x PC5	45.66	0.19	0.33	0.54	0.06	1.97	0.12	1.27	97	96	13	21	3	4
PC2 x PC3	45.60	0.20	0.33	0.52	0.03	2.73	0.09	1.44	98	98	3	4	2	2
PC2 x PC4	35.76	0.40	0.01	1.83	0.02	3.10	0.03	2.07	96	96	3	4	4	4
PC2 x PC5	28.77	0.34	0.001	2.13	0.01	3.17	0.03	2.24	95	95	1	2	5	5
PC3 x PC4	26.33	0.12	0.85	0.89	0.18	0.87	0.52	0.15	99	99	7	10	1	1
PC3 x PC5	19.34	0.17	0.68	0.56	0.11	1.48	0.46	0.27	99	98	8	10	1	2
PC4 x PC5	9.51	0.51	0.001	3.35	0.001	4.49	0.01	2.60	98	98	5	7	2	2

Table S3. Kernel density bandwidths of multidimensional hypervolumes of the four alien amphibian species.

	Bandwidth				
	PC1	PC2	PC3	PC4	PC5
Hylarana erythraea					
Native niche	0.49	0.52	0.55	0.20	-
Philippine niche	0.21	0.31	0.37	0.17	-
Rhinella marina					
Native niche	0.45	0.31	0.23	0.24	0.12
Philippine niche	0.35	0.35	0.29	0.48	0.12
Hoplobatrachus rugulosus					
Native niche	0.37	0.72	0.35	0.22	0.19
Philippine niche	0.27	0.75	0.34	0.22	0.19
Kaloula pulchra					
Native niche	0.50	0.33	0.65	0.19	0.07
Philippine niche	0.35	0.44	0.52	0.27	0.13

Supplementary methods

Data from Philippine-invaded range

Abantas, A. D. & Nuñeza, O. M. Species diversity of terrestrial vertebrates in Mighty Cave, Tagoloan, Lanao Del Norte, Philippines. *J. Biodiv. Envi. Sci.*, 5(6), 122–132 (2014).

Alcala, A. C. Philippine notes on the ecology of the Giant Marine Toad. *Silliman J.*, 6(2), 90–96 (1957).

Alcala, A.C. Amphibians of Negros Island including two new records. *Proc. Calif. Acad. Sci.*, 5(2), 171–174, (1958).

Alcala, M. L. R. et al. survey of the riparian vertebrate fauna of Señora River, Siquijor island, Central Philippines. *Silliman J.*, 52(2), 106–114 (2011).

Almeria, M. L. & Nuñeza, O. M. Amphibian diversity and endemism in the swamp forests of Agusan Marsh, Agusan del Sur, Philippines. *AES Bioflux*, 5(1), 30–48, (2013).

Alviola, P. A., Gonzales, J. C. T., Dans, A. T. L., Afuang, L. E. & Dimapilis, A. B. Herpetofauna of Puerto Galera, Mindoro Island, Philippines. *Sylvatrop*, 8(1&2), 86–93, (1999).

Ates, F. B. & Delima, E. M. M. Assemblage and microhabitats of anurans from Mt. Sinaka, Arakan, Cotabato and Mt. Hamiguitan, Davao Oriental, Mindanao Island, Philippines. *J. Nat. Stud.*, 7(1), 101–107 (2008).

Belleza, B. G. D. & Nuñeza, O. M. Herpetofaunal diversity and endemism in selected caves of Sarangani Province and Lanao del Sur, Philippines. *Adv. Environ. Biol.*, 8(21), 411–418 (2014).

Beukema, W. Herpetofauna of disturbed forest fragments on the lower Mt. Kitanglad Range, Mindanao Island, Philippines. *Salamandra*, 47(2), 90–98 (2011).

Boulenger, G. A. *Catalogue of the Batrachia Gradientia Salientia and S. E. Caudata in the Collection of British Museum*, ed. 2. (British Museum, 1882).

Brown, R. F., McGuire, J. A., Ferner, J. W., Icarangal, N. Jr. & Kennedy, R. S. Amphibians and reptiles of Luzon Island, II: preliminary report on the herpetofauna of Aurora Memorial National Park, Philippines. *Hamadryad*, 25(2), 175–195 (2000).

Brown, R. M, Ferner, F. W. Sison, R.V., Gonzales, J.C. & Kennedy, R.S. Amphibians and reptiles of the Zambales Mountains of Luzon Island, Republic of the Philippines. *Herpetol. Nat. Hist.*, 4(1), 1–22 (1996).

Brown, W. C. & Alcala, A. C. Comparison of the herpetofaunal species richness on Negros and Cebu Islands, Philippines. *Silliman J.*, 33(1-4), 74–86 (1986).

Brown, W. C. & Alcala, A. C. Populations of amphibians and reptiles in the submontante and montane forests of Cuernos De Negros, Philippines islands. *Ecology*, 42(4), 628–636 (1961).

Brown, W. C. & Alcala, A. C. The zoogeography of the herpetofauna of the Philippine Islands, a fringing archipelago. *Proc. Calif. Acad. Sci.*, 38(6), 105–130 (1970).

Brown, W. C. & Alcala. A. C. Observations on amphibians of the Mount Halcon and Mount Canlaon areas, Philippines Islands. *Silliman J.*, 2(2), 93–102 (1955).

Bucol, A. A., Alcala, E. L., Averia, L. T. & Cordova, L. The vertebrate biodiversity of the Gigantes and Sicogon Islands, Iloilo Province, Philippines. *Silliman J.*, 51(2), 105–131 (2010).

Bucol, A. A., Carumbana, E. E., & Averia, L. T. Status of the vertebrate fauna in selected sited of Pagatban River, Negros Oriental, Philippines. *Silliman J.*, 52(2), 91–105 (2011).

Causaren, R. M. Preliminary report on the anurans of Mts. Palay-Palay Mataas-Na-Gulod Protected Landscape, Luzon Island, Philippines. *Phil. J. Syst. Biol.*, 3, 40–56 (2009).

Delima, E. M. M., Ates, F. B. & Ibanez, J. C. Species composition and microhabitats of frogs within Arakan Valley Conservation Area, Cotabato, Mindanao Island, Philippines. Banwa, 3(1&2), 16–30 (2006).

Devan-Song, A. & Brown, R. M. Amphibians and reptiles of Luzon Island, Philippines, VI: the herpetofauna of the Subic Bay area. *Asian Herpetol. Res.*, 3(1), 1–20 (2012).

Diesmos, A. C., Brown, R. M. & Gee, G. V. A. Preliminary report on the amphibians and reptiles of Balbalasang-Balbalan National Park, Luzon Island Philippines. Sylvatrop, 13, 63–80 (2003).

Ferner, J. W., Brown, R. F., Sison, R. V. & Kennedy, R. S. The amphibians and reptiles of Panay Island, Philippines. *Asian Herpetol. Res.*, 9, 34–70 (2001).

Jose, R. P. Distribution of anuran species in Loboc Watershed of Bohol Island, Philippines. *Asian J. Biodiv.,* 3(86), 126–141 (2012).

Nuñeza, O. M. & Galorio, A. H. N. Cave hertpetofauna of Siargao Island Protected Landscape and Seascape, Philippines. *W. J. Environ. Biosci.*, 4(1), 26–35 (2015).

Nuñeza, O. M., Ates, F. B. & Alicante, A. A. Distribution of endemic and threatened herpetofauna in Mt. Malindang, Mindanao, Philippines. *Biodivers. Conserv*., 19, 503–518 (2010).

Oliveros, C. H., Ota, H., Crombie, R. I. & Brown, R. M. The herpetofauna of the Babuyan Islands, northern Philippines. *Scientific Papers, Natural History Museum, The University of Kansa*s, 43, 1–20 (2011).

Pagente, J. M. Q., Balaba, K. L. D., Peralta, S. A., Buenavista, D. P. & Sy, E. Y. Geographic distribution: *Kaloula pulchra* (Asiatic Painted Frog): Philippines, Mindanao Island, Bukidnon Province. *Herpetol. Rev.*, 47(2), 248 (2016).

Pedregosa-Hospodarsky, M., et al. *A faunal assessment of North Negros National Park (NNNP) Negros Island, Philippines.* Technical Report (The Rufford Small Grant Foundation, 2009).

Philippine National Museum of Natural History. *Herpetology Collection, Zoology Division*. Occurrence Dataset (PNMNH, 2019).

Rabor, D. S. & Alcala, A. C. Notes on a collection of amphibians from Mindanao Island, Philippines. *Phil. J. Sci*., 88(3), 334–344 (1959).

Ross, C. A. & Gonzales, P. C. Amphibians and reptiles of Catanduanes Island, Philippines. *Nat Mu. P*. (Manila), 2(2), 50–76 (1992).

Ross, C. A. & Lazell, J. D. Jr. Amphibians and reptiles of Dinagat and Siargao Island, Philippines. *Phil. J. Sci.*, 119(3), 257–285 (1990).

Schoppe, S. Key conservation amphibian species along Padanan Range, Northern Palawan, Philippines in *Conservation Studies on Palawan Biodiversity* (eds. Lacerna-Widmann, I.D., Widmann, P., Schoppe, S., van den Beukel, D. & Espeso, M.) (Katala Foundation, Inc., 2009).

Sison, R. V., Gonzales, P. C., & Ferner, J. W. New island records from Panay, Philippines. *Herpetol. Rev.*, 26(1), 48–49 (1995).

Soriano, P. Notes on the Food Habits of the Giant Toad (Bufo marinus Linn.) in the Philippines. *Phil. J. Plant Ind*., 29 (3-4), 79–86 (1964).

Sularte, R. P. et al. Species distribution and abundance of amphibians in two vegetation types of Agusan Marsh, Philippines. *AES Bioflux*, 7(1), 20–34 (2015).

Sy, E. Y. & Salgo, J. Geographic distribution: *Eleutherodactylus planirostris* (Greenhouse Frog): Philippines, Cebu Island. *Herpetol. Rev.*, 46(2), 212 (2015).

Sy, E. Y. et al. Geographic distribution: *Kaloula pulchra* (Asiatic Painted Frog): Philippines, Mindoro Island. *Herpetol. Rev.*, 47(2), 248 (2016).

Sy, E. Y. Geographic distribution: *Kaloula pulchra* (Asiatic Painted Frog): Philippines, Mindanao Island. *Herpetol. Rev.*, 44(4), 621 (2013).

Sy, E. Y., Eleazar, B. I., Achacoso, R. & Diesmos, A. C. Geographic distribution: *Eleutherodactylus planirostiris* (Greenhouse Frog): Philippines, Luzon Island. *Herpetol. Rev.*, 46(1), 56 (2015).

Sy, E. Y., Martyr, J. C. & Diesmos, A. C. Geographic distribution: *Eleutherodactylus planirostiris* (Greenhouse Frog): Philippines, Negross Island. *Herpetol. Rev.*, 46(1), 56 (2015).

Sy, E. Y., Villareal, D. &Gamolo, G. C. Geographic distribution: *Kaloula pulchra* (Asiatic Painted Frog): Philippines, Cebu Island. *Herpetol. Rev.*, 45(2), 276–277 (2014).

Sy, E.Y. & Malabana, D. A. O. Geographic distribution: *Kaloula pulchra* (Asiatic Painted Frog): Philippines, Mindoro Island. *Herpetol. Rev.*, 46(1), 212, (2015).

Sy, E.Y. Geographic distribution: *Eleutherodactylus planirostris* (Greenhouse Frog): Philippines, Palawan Island. *Herpetol. Rev.*, 48(3), 583 (2017a).

Sy, E.Y. Geographic distribution: *Eleutherodactylus planirostris* (Greenhouse Frog): Philippines, Mactan Island. *Herpetol. Rev.*, 48(3), 583 (2017b).

Taylor, E. H. Herpetological fauna of Mount Makiling. *Phil. Agriculturist*, 11(5), 127–139 (1922).

Turner, C., Tamblyn, A., Dray, R., Maunder, L. & Raines, P. *The biodiversity of the Upper Imbang-Caliban Watershed, North Negros Forest Reserve, Negros Occidental, Philippines*. (Coral Cay Conservation, 2003).
